# Particle-Based Modeling of Living Actin Filaments in an Optical Trap

**DOI:** 10.3390/polym8090343

**Published:** 2016-09-19

**Authors:** Thomas A. Hunt, Santosh Mogurampelly, Giovanni Ciccotti, Carlo Pierleoni, Jean-Paul Ryckaert

**Affiliations:** 1 D3-Computation, Istituto Italiano di Tecnologia, via Morego 30, Genova 16163, Italy; tomahunt@gmail.com; 2 Department of Chemical Engineering, University of Texas at Austin, Austin, TX 78712, USA; santoshcup6@gmail.com; 3 IAC, CNR, Via dei Taurini, Rome 00185, Italy; giovanni.ciccotti@roma1.infn.it; 4 Department of Physics, University of Rome “La Sapienza”, P.le Aldo Moro 2, Rome 00185, Italy; 5 School of Physics, University College Dublin, Belfield, Dublin 4, D04 V1W8, Ireland; 6 Department of Physical and Chemical Sciences, University of L’Aquila, Via Vetoio 10, L’Aquila 67100, Italy; 7 Department of Physics, Université Libre de Bruxelles (ULB), Campus Plaine, CP 223, Brussels B-1050, Belgium

**Keywords:** biofilaments, actin networks, molecular simulation

## Abstract

We report a coarse-grained molecular dynamics simulation study of a bundle of parallel actin filaments under supercritical conditions pressing against a loaded mobile wall using a particle-based approach where each particle represents an actin unit. The filaments are grafted to a fixed wall at one end and are reactive at the other end, where they can perform single monomer (de)polymerization steps and push on a mobile obstacle. We simulate a reactive grand canonical ensemble in a box of fixed transverse area *A*, with a fixed number of grafted filaments $N_{f}$, at temperature *T* and monomer chemical potential $\mu_{1}$. For a single filament case ($N_{f}=1$) and for a bundle of $N_{f}=8$ filaments, we analyze the structural and dynamical properties at equilibrium where the external load compensates the average force exerted by the bundle. The dynamics of the bundle-moving-wall unit are characteristic of an over-damped Brownian oscillator in agreement with recent in vitro experiments by an optical trap setup. We analyze the influence of the pressing wall on the kinetic rates of (de)polymerization events for the filaments. Both static and dynamic results compare reasonably well with recent theoretical treatments of the same system. Thus, we consider the proposed model as a good tool to investigate the properties of a bundle of living filaments.

## 1. Introduction

When a bundle of parallel actin filaments in supercritical conditions hits a moving wall subject to an opposing constant load force $F_{L}$, the balance between the polymerization force, the load and the friction force from the solvent (usually negligible) leads to a stationary velocity *v* of the obstacle. Here, *v* indicates a coarse-grained velocity averaged over rapid fluctuations due to microscopic events, like the addition/removal of single monomers occurring at the bundle tip and to the usual wall Brownian motion. A stationary non-equilibrium state is only possible if the living filaments are rigid so that the distribution of the tip-wall distance(s) (for single or many filaments) becomes independent on the length of the bundle [1,2,3,4] and also independent of time in a time window much longer than the obstacle diffusion time and the chemistry time (inverse polymerization rate). When filament flexibility is taken into account, both the filament length and the wall position (with respect to the origin of the filaments) become relevant variables, and the dynamical state can no longer be stationary, not even on a coarse-grained time window [5,6,7].

For rigid living polymers pressing against a mobile wall, the Brownian ratchet (BR) model, in the fast wall-diffusion limit, provides the simplest theoretical rationalization of the conversion of chemical energy into mechanical work against the load [1]. A single rigid filament originating from a grafted seed, subjected to polymerization and depolymerization steps with size increment *d* at its unique active end, is perturbed by a fluctuating moving wall. Any polymerization attempt (with attempt rate ${\tilde{U}}_{0}$) is rejected if it leads to overlap with the wall, but is accepted otherwise. The depolymerization steps occur at a uniform rate ${\tilde{W}}_{0}$, independently of the tip-wall distance. The wall is undergoing a 1D drifted Brownian motion limited on one side by the rigid filament tip and otherwise characterized by a friction coefficient $\zeta^{\prime }$, a temperature *T* and a systematic force term $F_{L}$ directed towards the filament’s seed. If the friction is low, $\beta \zeta^{\prime }d^{2}\ll \min\left[{\tilde{U}}_{0}^{-1},{\tilde{W}}_{0}^{-1}\right]$ (where $\beta =1/k_{B}T$), the fast Brownian motion of the wall leads rapidly to a stationary distribution $P\left(x\right)\propto \exp\left(-\beta F_{L}x\right)$ in terms of the tip-wall distance *x*, between any pair of successive chemical events. The resulting stationary velocity is [1]:

(1) $$v\left(F_{L}\right)=d\;\left[{\tilde{U}}_{0}\;e^{-(d\;F_{L}/k_{B}T)}-{\tilde{W}}_{0}\right],$$

where the exponential term gives the probability for an attempted polymerisation step to be successful or equivalently the probability for the wall to create a gap of size *d*. No closed expression of the velocity $v(F_{L};\zeta^{\prime })$ exists when the wall diffusion gets slower (as $\zeta^{\prime }$ gets larger), but it generally leads to a slight decrease of the velocity [8,9,10]. Generalization of this single rigid living filament model towards many filament bundles has been developed over the years [2,3,11], and the exact form of the stationary velocity $v(F_{L};N_{f})$ for a homogeneous bundle of $N_{f}$ filaments with staggered seeds in the high diffusion limit has been established recently [2,4].

Bundles growing against membranes have also been simulated on the basis of *ad hoc* stochastic models [12,13] where the obstacle to polymerization is a fluctuating membrane. Filaments are treated as living rigid cylinders, which, in the latter case, have an excluded volume repulsion for the diffusing monomers, which are treated explicitly. Therefore, in the vast majority of applications where an actin bundle is pushing on a mobile obstacle, the filaments are assumed to be rigid.

When the filament’s flexibility is taken into account, the situation is much more complex as both the fluctuations of the wall position and the tip bending fluctuations must couple to the chemical steps [3]. Flexible filaments pushing against a mobile wall subject to constant load were investigated recently by dynamic Monte Carlo [8,9]. In this simulation study, the filaments have bending degrees of freedom and can grow or shrink with respective probabilities ${\tilde{U}}_{0}$ and ${\tilde{W}}_{0}$, the acceptance of a polymerization step being conditioned by a criterion of no overlap with the wall. The wall is undergoing a biased random walk as in the BR model. Emphasis was put on the effects of flexibility and wall diffusivity on the pseudo-stationary velocity $v(F_{L};L,\zeta^{\prime })$ of a single filament in supercritical conditions where polymerization steps dominate. The *L* dependence of $v(F_{L};L,\zeta^{\prime })$ for cases with finite persistence length $L_{p}$ was not explicitly investigated. The authors stress however the conditions on *L* and $F_{L}$ to observe the lateral escape of the polymerizing filament: the spectacular fluctuation, which they called pushing catastrophe, becomes possible when a bending fluctuation allows the filament to start growing parallel to the obstacle, preventing further conversion of chemical energy into mechanical work against the load.

**Figure 1:**
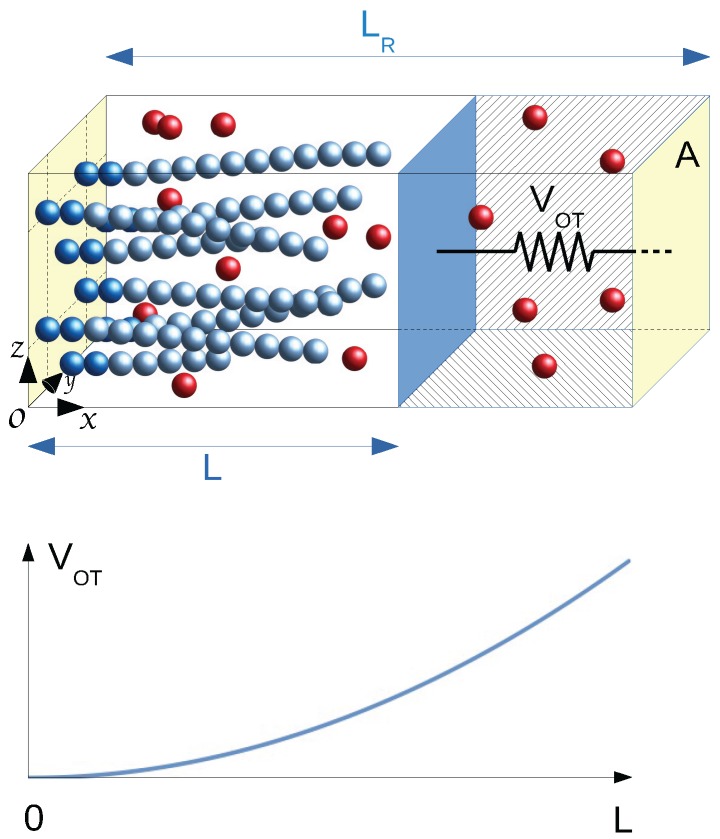
The reactive grand canonical ensemble considers the box of size $A\times L_{R}$ limited by two solid walls at x=0 and at $x=L_{R}$ represented by planes in yellow, with fixed temperature *T* and fixed free monomer chemical potential $\mu_{1}$. The system, periodic in the *y* and *z* directions, is divided into two chambers by a mobile wall (depicted as the plane in dark blue) oriented parallel to the fixed walls and located at the variable x=L position ($0\leq L\leq L_{R}$). The filament seeds (two first beads in dark blue) are grafted in the wall at x=0 (on a centered square lattice for the illustrated case $N_{f}=8$), and filaments (variable part represented by light blue beads) grow in supercritical conditions within the left chamber at the expense of free monomers (red beads). In the second chamber, only free monomers are present at the imposed chemical potential $\mu_{1}$. The mobile wall is loaded by a restoring trap force $\kappa_{T}L$ indicated by the compressed spring symbol drawn in the right chamber, having a stored mechanical energy $V_{\mathrm{OT}}=\frac{1}{2}\kappa_{T}L^{2}$ represented in the graph below. Equilibrium results from the cancellation of the sum of the *L* dependent restoring force and the bundle polymerization force. The molecular dynamics simulation treats explicitly the left chamber system only, the action on the moving wall by the free monomers in the right chamber being replaced by the average force $p(\mu_{1},T)A$ parallel to the load force where *p* is the pressure of a free monomers gas at ($\mu_{1},T$). The wall dynamics and the individual monomer dynamics (both the free ones and those integrated into filaments) are described by Langevin dynamics assuming a solvent producing free draining with friction coefficients $\zeta^{\prime }$ for the wall and *ζ* for monomers. Instantaneous (de)polymerizing reactions follow a local Monte Carlo algorithm integrating or rejecting a free monomer at one filament tip. Free monomer chemical potential $\mu_{1}$ is maintained in the simulation box by the addition/deletion of free monomers.

To analyze the specific features of flexibility in living biofilaments, it is advantageous to replace the constant load setup by a space-dependent load leading to the establishment of a true equilibrium state. In a series of two papers [5,6], we have established the statistical mechanics framework of a bundle of living filaments in chemical equilibrium with free monomers, i.e., a reactive grand canonical ensemble (GC), within a two-chambered box mimicking an optical trap apparatus (see Figure 1). The bundle with a fixed number $N_{f}$ of living filaments grafted to the fixed wall of the first chamber presses against a mobile wall subject to an harmonic restoring force $-\kappa_{T}L$ where the distance *L* is measured from the grafting wall. The whole box of fixed length $L_{R}$ and fixed transverse area *A* is coupled to a thermostat at temperature *T*, and both chambers, separated by the mobile partitioning wall ($0<L<L_{R}$), contain free monomers at the same imposed chemical potential $\mu_{1}>\mu_{1c}$ where $\mu_{1c}$ is the value of the chemical potential at the critical state defined as the state where the filament in the bulk has no tendency either to grow or to shrink. By tuning the trap strength $\kappa_{T}$ at fixed ($N_{f},L_{R},A,\mu_{1},T)$, one can study the equilibrium properties of bundles of filaments for different (self-adjusted) average positions of the wall. The average optical trap length 〈L〉 for bundles of flexible filaments in ideal conditions can be roughly estimated by Hill’s polymerization force value $F_{H}$ [14,15,16], the exact average force for rigid filaments [6], so that we expect [5,6]:

(2) $$\langle L\rangle \approx \frac{F_{H}}{\kappa_{T}}=\frac{k_{B}T}{d\;N_{f}\;\kappa_{T}}\ln{\hat{\rho }}_{1}$$

where:

(3) $${\hat{\rho }}_{1}=\exp\left(\beta (\mu_{1}-\mu_{1c})\right)=\frac{{\tilde{U}}_{0}}{{\tilde{W}}_{0}}>1$$

is the free monomer density reduced by its critical value.

In this paper, we exploit a 3D coarse-grained particle-based model (adapted from [17]), and we follow explicitly the 1D dynamics of the wall, of the grafted bundle filaments, consisting of linear assemblies of connected monomers, and of the diffusing free monomers, while the system is subjected to explicit reactive (de)polymerizing events modifying continuously the size of the filaments. To illustrate our simulation approach at fixed temperature *T* and at fixed chemical potential $\mu_{1}$, we treat two cases: a single grafted semi-rigid filament and a $N_{f}=8$ filaments bundle, pressing against a mobile wall subject to a harmonic restoring potential. The dynamics of the wall and of all monomers (both free monomers or monomers integrated into filaments) includes the solvent by a Langevin approach with monomer and wall friction coefficients *ζ* and $\zeta^{\prime }$, respectively. The free monomer chemical potential is maintained by homogeneously adding/removing particles using a Widom insertion scheme. A Monte Carlo local move, with the attempt rate using Poisson reaction time statistics and the acceptance rate based on energetic criteria, deals with reactive monomer assembly/disassembly. The reaction scheme satisfies the detailed balance and leads to chemical equilibrium between the free monomers’ concentration and the filament size populations, strongly influenced by the wall pressure on the filament. The average force and the average number of hitting filaments and their fluctuations are computed and analyzed in reference to theoretical predictions proving that our present model reproduces the equilibrium properties of the system well [6]. More importantly, the fluctuations of the wall position and of the filament size show that the system behaves dynamically as a Brownian damped oscillator where the friction term contains both the hydrodynamic component from the solvent and a chemical component related to the filament size changes, with an amplitude [6] of the order of:

(4) $$\sigma_{L}\equiv \sqrt{\langle {\left(L-\langle L\rangle \right)}^{2}\rangle }=\sqrt{k_{B}T/\kappa_{T}}$$

which corresponds typically to a few monomer sizes *d* in the actin case. We provide evidence that the chemical friction is originated by the BR mechanism invoked in the theoretical treatment of the system. In this work, we limit ourselves explicitly to rather rigid filaments, therefore limiting the effects of flexibility, with the specific aim to validate the various aspects of the model against known results. The properties of longer and more flexible bundles will be investigated in a future work.

The paper is organized as follows. In Section 2, we describe the microscopic model for our reactive mixture of grafted filaments and free monomers. In Section 3, we analyze the various time scales for an actin bundle in the optical trap experiment. We also explain how we set up our model and discuss the choice of parameters that are adopted in the simulation. In Section 4, we present static and dynamic results for a single filament ($N_{f}=1$) and for a homogeneous bundle with eight filaments ($N_{f}=8$). Our conclusions and perspectives are gathered in the last Section 5. Details of the modeling of the chemical steps and the imposing of the free monomer chemical potential are provided in the Appendix A and Appendix B, respectively.

## 2. Microscopic Model of the System in an Optical Trap Setup

Our simulations deal with actin proteins, modeled as spherical units, which can be either free monomers or part of a grafted F-actin filament, a semi-flexible linear assembly of monomers. The system, illustrated in Figure 1, is enclosed in a rectangular box of length $L_{R}$ along the *x* laboratory axis, having a square transverse area $A={L^{\prime }}^{2}$ (in the yz−plane). Periodic boundary conditions apply in transverse directions only. The box is limited in the *x* direction by two fixed walls located at $x_{w}=0$ and $x_{w}=L_{R}$. This box is divided into two chambers of volumes LA for Chamber 1 and $(L_{R}-L)A$ for Chamber 2, separated by an additional mobile transverse partition wall of area *A*, localized by a variable position *L* along *x* ($0<L<L_{R}$), denoted as the mobile wall.

This two-chamber structure allows us to mimic the experimental setup of an actin bundle in an optical trap [18]. We consider an actin bundle with a fixed number $N_{f}$ of filaments, grafted in the fixed wall of Chamber 1 and in contact with a bath of reacting free monomers, pressing against the moving wall (the colloidal particle trapped within an optical trap). In Chamber 2, a bulk solution of free monomers in contact with the same free monomer bath is pressing on the opposite side of the moving wall. All interacting units and the mobile wall (bead) are embedded in a viscous solvent, which will be included in the particle and wall’s equations of motion according to the classic Langevin approach. Importantly, the mobile wall is further subject to an external load taken as a harmonic restoring force $-\kappa_{T}L$ where $\kappa_{T}$ is the trap force constant. All forces applied to the moving wall appear in a 1D Langevin equation dealing with the wall dynamics, directly coupled to the monomer dynamics. The two chambers are directly connected to a common thermostat (Langevin bath at fixed temperature) and to a common reservoir of free monomers at fixed chemical potential $\mu_{1}$, which can exchange free monomers with both Chamber 1 and Chamber 2.

The dynamics of monomers is coupled to instantaneous single monomer (de)polymerization reactions taking place locally at the filament tips, thus only in Chamber 1. In supercritical conditions, the filaments tend to grow and, thus, finally press on the mobile wall. Thanks to the opposite load force increasing with *L*, a stable equilibrium (including chemical equilibrium) is attained when the wall position oscillates around a stable value: we are then sampling the optical trap ensemble for a bundle of living filaments pressing against a mobile wall, within the framework of the reactive grand canonical ensemble [6].

Although the simulation could include the two coupled subsystems occupying the two chambers, we have restricted our microscopic simulations to the primary box only. The pressure exerted by the free monomers in the secondary chamber is taken into account by adding an average pressure force pA on the mobile wall, where $p=p(\mu_{1},T)$ is the pressure of a pure free monomer solution at the imposed chemical potential $\mu_{1}$ and temperature *T*. Pressure force fluctuations on the moving wall due to free monomers in the secondary box are thus neglected.

In the following subsections and in the two Appendices, we detail the model of the reactive mixture embedded in Chamber 1.

### 2.1. The Actin Bundle-Free Monomer Reactive Mixture

The $N_{f}$ actin filaments in the reacting chamber are grafted normally to the fixed wall at $x_{w}=0$. Free monomers are present in the same volume, and their number fluctuates in time because of chemical reactions with the filaments and because of the exchange of particles with the reservoir at fixed free monomer chemical potential $\mu_{1}$. Filaments are thus allowed to grow (shrink), their contour length $L_{c}$ changing by *d* (monomer size in F-actin) each time a monomer is incorporated into (removed from) the linear structure. They can exert a direct force on the mobile wall when their contour lengths approach the distance ≈L between the two walls.

Let $N_{t}\left(t\right)$ be the total number of monomers in the simulation box at some time *t*. We will need to trace not only the monomers’ positions, but also their insertion/deletion for grand canonical statistics and their chemical character, since they can continuously interchange between free and assembled monomers. A pair of dynamical topological indices (n,k) are assigned to each monomer in the system. The first index $n\in [0,N_{f}]$ indicates the specific filament, n=0 meaning that the monomer belongs to the solute bath. The second index $k\in [1,j_{n}]$ indicates the rank position of the monomer in the specific filament *n*. Monomers with indices k=1 and k=2 are the first two permanent and fixed monomers forming the seed and used to graft the filaments to the wall. Monomers with $k=j_{n}\left(t\right)$ represent the tip monomer of filament *n* at time *t*. For n=0, $j_{0}\left(t\right)$ represents the number of free monomers in the system at that time; hence $N_{t}\left(t\right)=\sum_{n=0}^{N_{f}}j_{n}\left(t\right)$. Index $j_{0}\left(t\right)$, hence $N_{t}\left(t\right)$, fluctuates because of the coupling to an external solute bath at fixed free monomer chemical potential $\mu_{1}$ [6]. Between single particle exchange events, $N_{t}$ is constant, but the chemical character and, therefore, the topological indices of the monomers can change because of the occurrence of chemical events. The first two monomers of each filament, (n,1) and (n,2), have fixed coordinates $\left(h_{n},y_{n},z_{n}\right)$ and $\left(h_{n}+d,y_{n},z_{n}\right)$, respectively. Any particular filament length $j_{n}$ is restricted to $j_{n}\geq 2$. In the special case of a single filament ($N_{f}=1$), we set $h_{n}=0$ while the $y_{n}$ and $z_{n}$ seed coordinates are irrelevant given the translation symmetry in the transverse plane. For $N_{f}>1$, we locate the position of filament seeds in the grafting plane on a planar body-centered square lattice of unit cell size $a=L^{\prime }/l$ where *l* is an integer. Choosing $N_{f}=2l^{2}$, the surface density (transverse area per filament) is ${\left(L^{\prime }\right)}^{2}/\left(2l^{2}\right)$. The longitudinal disposition of the filament is of primary importance [6]. Setting $h_{n}=0$ for all *n* defines a bundle in the registry (unstaggered). In our application to a multi-filaments bundle, we will adopt the homogeneous bundle case (staggered disposition of seeds) setting, for *n* even, $h_{n}=\left(\frac{n-0.5}{N_{f}}-0.5\right)d$.

### 2.2. The Potential Field

The potential field employed comprises the intrafilament part and the monomer-wall part, since we limit the present study to ideal gas conditions. However a term for monomer-monomer interactions could be included easily.

The intrafilament part for a filament of $j_{n}$ monomers is a sum of $\left(j_{n}-2\right)$ stiff bonding potentials $U^{b}\left(r\right)$ forcing the distance *r* between adjacent monomers (with logical indices (n,k) and (n,k+1)) to remain close to *d* (the first grafted bond is rigid with size *d*). The filament persistence length $L_{p}$ is imposed via a sum of $\left(j_{n}-2\right)$ three body bending terms $U^{\mathrm{bend}}\left(\theta \right)$ implying the bending angle *θ* between adjacent bonds (i.e., the three monomers with topological indices (n,k),(n,k+1),(n,k+2)). Explicitly, we have taken:

(5) $$\begin{array}{cc} U^{b}\left(r\right) & =-\epsilon_{0}+\frac{1}{2}k_{s}{\left(r-d\right)}^{2} \end{array}$$

(6) $$\begin{array}{cc} U^{\mathrm{bend}}\left(\theta \right) & =\kappa [1-\cos\left(\theta \right)] \end{array}$$

with $\kappa =5370k_{B}T$ and $k_{s}=4000k_{B}T/d^{2}$. We first note that, when *d* is set to the monomer size (d=2.7nm), the chosen value of $\kappa =L_{p}k_{B}T/d$, for $k_{B}T=1$, leads to the typical F-actin experimental value $L_{p}=5370\times 2.7\;\mathrm{nm}=14.5\;\mu m$ [19]. As for the bonding spring constant $k_{s}$, we notice that an F-actin filament of one micron length has a stiffness to longitudinal compression of (44±5)pN/nm (see Footnote b of Table 8.1 in Reference [19]). A fragment of length d=2.7nm has thus a stiffness of 16.28N/m (Here, we use the composition relation for 1000/2.7≃370 identical springs of rest length *d* in series: $k_{\mathrm{tot}}^{-1}=\sum_{i}\;k_{i}^{-1}$). As the force constant unit is $k_{B}T/d^{2}=0.57\;\mathrm{pN}/\mathrm{nm}$, one gets $k_{s}=$ (29,000 $\;\pm \;1500)k_{B}T/d^{2}$. Our choice to take a value of $k_{s}$, which is quite a bit lower, is dictated by the need to avoid very fast intramolecular modes, which would require a small time step in the numerical integration of the equations of motion.

Like in a previous study [20], we adopted a purely repulsive 9-3 potential for the monomer-wall interaction:

(7) $$\begin{array}{cc} U^{w}\left(s\right) & =\epsilon_{w}\left[\frac{3\sqrt{3}}{2}\left[{\left(d/s\right)}^{9}-{\left(d/s\right)}^{3}\right]+1\right]\;\;\;\mathrm{if}\;s\leq s_{c}. \end{array}$$

(8) Uw(s)=0ifs>sc.332[(d/s)9−(d/s)3]+1

where $s=\left|x-x_{w}\right|$ and $x_{w}=0,L$ corresponds to the position of grafting and the moving wall, respectively, and $\epsilon_{w}=0.1k_{B}T$. The wall potential is truncated beyond its minimum at $s_{c}=3^{1/6}d$, where it vanishes.

The constant $\epsilon_{0}=13.644143k_{B}T$ in Equation (5) represents the energy gain as a new bond is created within the filament during a polymerization step (and conversely, an energy loss when a bond is removed during a depolymerization step) [5]. As shown in [21], this quantity enters in the size independent bulk equilibrium constant $K_{0}$ of the (de)polymerization chemical reaction, established by the chemical equilibrium condition $\mu_{i+1}=\mu_{i}+\mu_{1}$, where $\mu_{i}$ (i≥2) indicates the chemical potential of a filament of *i* monomers. In ideal conditions, the equilibrium free monomer density $\rho_{1}$ and the distribution $P_{i}$ of filaments of size *i* are linked by [5,6,22]:

(9) $$\begin{array}{c} \frac{P_{i+1}}{P_{i}\rho_{1}}=\frac{q_{i+1}}{q_{i}q_{1}/V}\equiv K_{0} \end{array}$$

where the *q*’s are a single grafted filament or free monomer canonical partition functions and *V* is the system volume. For the intramolecular model defined by Equations (5) and (6), we established in [21] that:

(10) $$\begin{array}{c} K_{0}=\exp\left(\beta \epsilon_{0}\right)\frac{2\pi d^{4}}{L_{p}}{\left(\frac{2\pi }{\beta k_{s}d^{2}}\right)}^{1/2}\left[1-\exp\left(-2L_{p}/d\right)\right]. \end{array}$$

Given the adopted values of the parameters, Equation (10) gives $K_{0}=39.07144d^{3}$. At the critical state $P_{i+1}/P_{i}=1,\forall i\geq 2$, and Equation (9) provides:

(11) $$\begin{array}{cc} \rho_{1c} & =1/K_{0}=0.025594d^{-3} \end{array}$$

which is the value of the critical density of free monomers corresponding to a critical chemical potential:

(12) $$\begin{array}{cc} \mu_{1c} & =k_{B}T\ln\left(\rho_{1c}\Lambda^{3}\right) \end{array}$$

where Λ is the de Broglie thermal length of the free monomers. We define a convenient effective chemical potential $\mu_{1}^{*}$ as:

(13) $$\begin{array}{c} \mu_{1}^{*}\equiv \mu_{1}-k_{B}T\ln{\left(\Lambda /d\right)}^{3}=k_{B}T\ln\left(\rho_{1}d^{3}\right) \end{array}$$

Equation (11) provides:

(14) $$\begin{array}{cc} \mu_{1c}^{*} & =k_{B}T\ln\left(\rho_{1c}d^{3}\right)=-3.6654k_{B}T. \end{array}$$

as a critical value of the effective chemical potential.

### 2.3. Monomer and Wall Equations of Motion.

The dynamics of each monomer *i* (here, *i* is a short notation for the pair of indices (n,k)) is described by a Langevin equation:

(15) $$M\frac{d^{2}r_{i}}{dt^{2}}=\left[F_{i}^{\mathrm{intra}}+F_{i}^{\mathrm{inter}}+F_{i}^{w}\right]-\zeta \frac{dr_{i}}{dt}+S_{i}$$

where *M* is the monomer’s mass and *ζ* the solvent friction coefficient with associated random force $S_{i}$. $F_{i}^{\mathrm{intra}}$ is the sum of intrafilament forces imposing linear connectivity and bending flexibility, only present for monomers (n,k) with n>0. $F_{i}^{\mathrm{inter}}$ is the excluded volume (EV) term. Our theoretical developments consider explicitly EV terms even if we have disregarded them in the applications presented in the Result section. Finally, $F_{i}^{w}$ is the interaction of monomer i=(n,k) with the confining walls in the $\hat{x}$ direction.

The wall dynamics follows the 1D Langevin equation:

(16) $$M_{w}\frac{d^{2}L}{dt^{2}}=\left[F_{w}^{\mathrm{bun}}+F_{w}^{m}\right]-\kappa_{T}L-pA-\zeta^{\prime }\frac{dL}{dt}+R$$

where $M_{w}$ is the mass of the wall and $\zeta^{\prime }$ the solvent friction coefficient on the moving wall with associated random force *R*. $F_{w}^{\mathrm{bun}}$ is the total force on the wall due to the grafted filaments, and $F_{w}^{m}$ is the total force exerted by free monomers. pA is the average pressure term applied to the moving wall due to the free monomers in Chamber 2 of Figure 1 with $p=p(\mu_{1},T)$.

The fluctuation dissipation theorem requires that the random forces $S_{i}\left(t\right)$ and R(t) satisfy:

(17) $$\begin{array}{cc} \langle S_{i}\left(t\right)\rangle & =0;\;\;\;\langle S_{i}\left(t+t^{\prime }\right)\cdot S_{i}\left(t^{\prime }\right)\rangle =6\zeta k_{B}T\delta \left(t\right) \end{array}$$

(18) $$\begin{array}{cc} \langle R(t)\rangle & =0;\;\;\;\langle R\left(t+t^{\prime }\right)R\left(t^{\prime }\right)\rangle =2\zeta^{\prime }k_{B}T\delta \left(t\right) \end{array}$$

which sets up the temperature *T* of the thermal bath.

Since we will consider the case of no excluded volume, we have:

(19) $$\begin{array}{c} p\left(\mu_{1},T\right)=\frac{k_{B}T}{\Lambda^{3}}\exp\left(\mu_{1}/k_{B}T\right)=\frac{k_{B}T}{d^{3}}\exp\left(\mu_{1}^{*}/k_{B}T\right) \end{array}$$

### 2.4. Numerical Integration of the Equations of Motion

The monomer and wall equations of motion, Equations (15) and (16), are stochastic second order differential equations. To numerically integrate those equations, we exploited the algorithm proposed by Vanden-Eijnden and Ciccotti [23] (Equation (23) in [23]).

For a one-dimensional system with velocity *v* and position *x*, the integrator reads:

(20) $$\begin{array}{cc} v^{n+1/2} & =v^{n}+\frac{1}{2}\sqrt{h}\sigma \phi^{n}+\frac{1}{2}h\left(f\left(x^{n}\right)-\gamma v^{n}\right)\left(1-\frac{h\gamma }{4}\right)-\frac{1}{4}h^{3/2}\gamma \sigma \left(\frac{1}{2}\phi^{n}+\frac{1}{\sqrt{3}}\eta^{n}\right) \end{array}$$

(21) $$\begin{array}{cc} x^{n+1} & =x^{n}+hv^{n+1/2}+h^{3/2}\sigma \frac{1}{2\sqrt{3}}\eta^{n} \end{array}$$

(22) $$\begin{array}{cc} v^{n+1} & =v^{n+1/2}+\frac{1}{2}\sqrt{h}\sigma \phi^{n}+\frac{1}{2}h\left[f\left(x^{n+1}\right)-\gamma v^{n+1/2}\right]\left(1-\frac{h\gamma }{4}\right)-\frac{1}{4}h^{3/2}\gamma \sigma \;\left(\frac{1}{2}\phi^{n}+\frac{1}{\sqrt{3}}\eta^{n}\right) \end{array}$$

Here, *γ* is the friction coefficient divided by the mass, $\phi^{n}$ and $\eta^{n}$ are independent Gaussian variables with zero mean and unitary variance and $\sigma =\sqrt{2k_{B}T\gamma /m}$. We have adopted a time step of $h=5.33\times 10^{-5}\tau_{D}$ required to cope with the vibrational frequencies of our model.

### 2.5. The Free Actin Chemical Potential

The system is surrounded by a reservoir of free monomers at fixed effective chemical potential $\mu_{1}^{*}$ or equivalently at fixed bulk reduced density ${\hat{\rho }}_{1}$:

(23) $$\begin{array}{c} \ln{\hat{\rho }}_{1}=\beta \left(\mu_{1}^{*}-\mu_{1c}^{*}\right) \end{array}$$

obtained from Equations (13) and (3).

We control $\mu_{1}^{*}$ by adding/removing free monomers along the dynamical trajectory of the system. These addition and removal attempt steps, chosen with equal probability, are performed at randomly-selected Poisson times with adjustable rate $\nu_{\mathrm{GC}}$, using a standard algorithm [24]. We used a version with the extra condition that if the added or removed free monomer turns out to be chemically reactive (susceptible to polymerize with a filament tip), the move is automatically refused. Appendix B provides the justification and detailed procedure. In the present case of a confined ideal system, we get an effective rate of acceptance of the order of 90%.

### 2.6. The (De)Polymerization Steps

Our system is reactive with grafted filaments subject to micro-reversible (de)polymerization steps involving the release or the capture of one free monomer at the filament reactive end. A detailed account of the procedure to perform these (de)polymerization steps and its justification are given in Appendix A.

## 3. Numerical Experiments versus Real Experiments

We exploit the, so far unique, experimental results on an actin bundle in an optical trap [18] to estimate all experimental parameters and relevant length and time scales. The complexity of the system under consideration induces a number of widely-separated (many orders of magnitude) characteristic time scales. We need to contract this separation considerably in order to capture all relevant time scales in the same simulation. We argue that this does cause major problems as far as the order of the various characteristic times is respected, and their separation is large enough. We select the set of parameters gathered in Table 1 in order to reproduce, as best as possible, the experimental situation [18]. In the following, we justify our choices.

We select the monomer size *d*, the thermal energy $k_{B}T$ and the wall diffusion time:

(24) $$\tau_{D}=d^{2}/D$$

the time needed for the colloidal bead with diffusion constant *D* to diffuse in pure water solvent over a distance *d*, as units of length, energy and time, respectively. The monomer size in the F-actin filament is d=2.7 nm; the thermal energy at T=300K is $k_{B}T=4.142\times 10^{-21}$ J. The unit of time $\tau_{D}$ for the experimental system can be obtained by estimating the diffusion through the friction experienced by a colloidal bead of radius R=1μm in pure water (η≃0.001Pas). By the Stokes law, the friction is $\zeta^{\prime }=6\pi \eta R=1.9\times 10^{-8}{\mathrm{Js}/m}^{2}$. From $D=k_{B}T/\zeta^{\prime }$, we obtain the value $\tau_{D}=d^{2}\zeta^{\prime }/k_{B}T=3.5\times 10^{-5}$ s, reported in Table 1.

The persistence length $L_{p}/d$ in the model is fixed to a typical experimental value $L_{p}=14.5\mu$m. Another relevant length scale is the typical absolute length of (un-crosslinked) actin filaments pressing on the wall. The equilibrium value 〈L〉/d is roughly given by Equation (2) in terms of the external parameters $N_{f}$, ${\hat{\rho }}_{1}$ (or equivalently $\mu_{1}^{*}$) and $\kappa_{T}$ [6]. To remain in the non-escaping regime where the obstacle can effectively stop polymerization, 〈L〉/d must remain in the range 20–100 for the range of ${\hat{\rho }}_{1}$ explored [6]. Hence, all external parameters are adjusted in the simulations to cope with the experimental situation. We thus note that all length scales probed in our mesoscopic simulations are representative of corresponding experimental quantities.

$K_{0}d^{-3}$ is the bulk equilibrium constant of the (de)polymerization reaction, which, using Equation (11), fixes the free monomers’ critical concentration $\rho_{1c}$. The physically relevant quantity is, however, ${\hat{\rho }}_{1}=\rho_{1}/\rho_{1c}$, the reduced free monomer density, which lies in the range 1.5–4.0 in most in vitro experiments. This quantity is directly linked to $\mu_{1}^{*}$ using Equation (3), that is the chemical free energy, which can be converted into useful mechanical work by compressing the trap. The optical trap strength used in experiments is of the order of $\kappa_{T}\approx 0.008\;\mathrm{pN}/\mathrm{nm}$ [18]. As shown in [6], in the present range of values of ${\hat{\rho }}_{1}$, we need to use trap strengths per filament in the range $\kappa_{T}/N_{f}\geq 0.017$ pN/nm to avoid the escaping filament regime, of the same order of magnitude as in the experiments. Energy scales, length scales and, thus, typical trap and polymerization forces are realistic in our simulations.

We now investigate the much more complex situation with time scales. All of the relevant ones are gathered in Table 2 and discussed below as relative quantities with respect to $\tau_{D}$ defined earlier in Equation (24).

### 3.1. Experimental vs. Model Time Scales

In this mesoscopic system, there are a number of characteristic time scales that need to be considered for realistic modeling. From fast to slow modes, they are: the intrafilament modes, bond stretching, $\tau_{s}$, and bond bending, $\tau_{b}$; the free monomer characteristic times, inertial and diffusion times, $\tau_{\mathrm{fm}}^{\mathrm{in}}$ and $\tau_{\mathrm{fm}}$, respectively; the obstacle characteristic times: inertial, oscillatory (due to the trap) and diffusion times, $\tau_{W}^{\mathrm{in}}$, $\tau_{T}$ and $\tau_{D}$, respectively, of the latex bead in the experiment (of the planar wall in our model); the characteristic time between two successive chemical events at the tip of the filaments $\tau_{\mathrm{chem}}$ in the bulk.

An estimate of the intramolecular times for F-actin can be obtained by assuming a bead-spring model similar to our present one. The characteristic times of the stretching and the bending modes are respectively $\tau_{s}=2\pi \sqrt{M/k_{s}}$ and $\tau_{b}=2\pi \sqrt{Md^{2}/2\kappa }$ where *M* is the mass of the bead (For the bending mode, we consider the single triatomic molecule with fixed central atom and fixed energy $E=\frac{1}{2}\kappa \theta^{2}+2\frac{1}{2}M{\left[d\dot{\theta }/2\right]}^{2}=C$). For actin M=42 kDa = 6.977 $\times \;10^{-23}$ kg, hence $\tau_{s}=12.9$ ps and $\tau_{b}=21.2$ ps using the values of $k_{s}=$ 29,000 $k_{B}T/d^{2}$ and $\kappa =5370k_{B}T$ (see Section 2.2). In terms of the obstacle diffusion time reported in Table 1 (our unit of time), we have $\tau_{s}=3.69\times 10^{-7}\tau_{D}$ and $\tau_{b}=6.06\times 10^{-7}\tau_{D}$.

In our simulation, we fixed $M=0.003556(\tau_{D}^{2}k_{B}T/d^{2})$, $k_{s}=4000k_{B}T/d^{2}$ and $\kappa =5370k_{B}T$ and obtain $\tau_{s}=5.9\times 10^{-3}\tau_{D}$ and $\tau_{b}=3.6\times 10^{-3}\tau_{D}$. Our choice of parameters provides intramolecular times, relative to $\tau_{D}$, roughly four orders of magnitude larger than in the experimental system. However, we believe that the remaining gap of three orders of magnitude is sufficient to decouple intramolecular modes from the slower modes in the system, still providing an affordable working framework. Furthermore, our choice of $k_{s}$ leads to contour length fluctuations about three times too large. Indeed, for a filament of N≃50 monomers, their amplitude is $\approx \sqrt{k_{B}TN/k_{s}}=0.1d$ (assuming *N* springs of stiffness $k_{s}$ in series and assuming that the average vibrational potential energy is $\left(1/2\right)k_{B}T$). Such small fluctuations should affect only marginally the rate of chemical (de)polymerization controlled by the wall position and the filament bending fluctuations.

The next characteristic times concern the free monomers, namely G-actin proteins in water solvent. The typical radius of a G-actin protein, considered as a spherical particle, is $r_{\mathrm{fm}}=2.9\mathrm{nm}=1.074d$ [25]. From the Stokes law, we get $\zeta =6\pi \eta r_{\mathrm{fm}}≃5.5\times 10^{-11}$ J s/m${}^{2}$. The inertial time of free monomer is thus $\tau_{\mathrm{fm}}^{\mathrm{in}}=M/\zeta ≃1.3\mathrm{ps}=3.6\times 10^{-8}\tau_{D}$ and the diffusion time $\tau_{\mathrm{fm}}=r_{\mathrm{fm}}^{2}/D_{\mathrm{fm}}=r_{\mathrm{fm}}^{2}\zeta /k_{B}T=1.1\times 10^{-7}s=3.14\times 10^{-3}\tau_{D}$, five orders of magnitude larger than $\tau_{\mathrm{fm}}^{\mathrm{in}}$.

In our simulation $r_{\mathrm{fm}}=d$, and we have chosen $\zeta =0.5\tau_{D}k_{B}T/d^{2}$, so that $\tau_{\mathrm{fm}}^{\mathrm{in}}=M/\zeta =7.1\times 10^{-3}\tau_{D}$ and $\tau_{\mathrm{fm}}=0.58\tau_{D}$. Here, the separation between the inertial and the diffusion time scales is only three orders of magnitude. More importantly, the relative diffusion time with respect to the obstacle diffusion time $\tau_{D}$ is quite a bit larger than in reality, meaning that free monomers diffuse much too slowly in our model. This could influence the rate of chemical events, hence the rate of supercriticality of the solution, since a chemical reaction needs available free monomers in the reacting region to occur. We obviate this deficiency by operating a continuous removal/addition of free monomers in the system in order to simulate the grand canonical ensemble at fixed $\mu_{1}^{*}$. We have adopted a rather high insertion/removal attempt frequency $\nu_{\mathrm{GC}}=187.5\tau_{D}^{-1}$ (see Section 2.5) to ensure a free monomer density in the system as uniform as possible. Since the total number of free monomers in the system $\langle N_{1}\rangle$ is of the order of 100 (see Table 3) and since the degree of acceptance of these moves is of the order of 90%, during the characteristic time $\tau_{D}$, each free monomer is inserted and removed once on average.

The next characteristic times concern the moving obstacle. In the absence of the F-actin bundle, the obstacle is subject to the harmonic potential from the optical trap and to the friction from the water solvent. For a harmonic oscillator with friction, the overdamped condition (real solutions of the characteristic equation) reads [26]:

(25) $$\begin{array}{c} {\left(\frac{M_{w}}{\zeta^{\prime }}\right)}^{2}={\left(\tau_{w}^{\mathrm{in}}\right)}^{2}\leq \frac{M_{w}}{4\kappa_{T}}=\frac{1}{16\pi^{2}}\tau_{T}^{2} \end{array}$$

where $M_{w}$ is the mass of the moving object and $\tau_{T}=2\pi \sqrt{M_{w}/\kappa_{T}}$ is the period of the obstacle oscillations in the optical trap. Assuming a mass $M_{w}=\frac{4\pi }{3}R^{3}\rho \approx 4.2\times 10^{-15}\mathrm{kg}$ for a latex bead of radius 1μm (we have assumed the density of latex roughly equal to the density of water) and the typical trap strength $\kappa_{T}=0.008\mathrm{pN}/\mathrm{nm}$ [18], the experimental values are $\tau_{w}^{\mathrm{in}}=2.23\times 10^{-7}s=6.3\times 10^{-3}\tau_{D}$ and $\tau_{T}=1.44\times 10^{-4}s=4.1\tau_{D}$.

In our model, we have chosen $M_{w}=0.010667\left(\tau_{D}^{2}k_{B}T/d^{2}\right)$ and $\zeta^{\prime }=\tau_{D}k_{B}T/d^{2}=1$; hence, $\tau_{w}^{\mathrm{in}}≃10^{-2}\tau_{D}$, $\tau_{T}=4.7\tau_{D}$ and $\tau_{T}=1.2\tau_{D}$ for the adopted $\kappa_{T}$ values, respectively $0.019375k_{B}T/d^{2}$ and $0.275k_{B}T/d^{2}$ for the $N_{f}=1$ and the $N_{f}=8$ cases.

This analysis shows that, both in the experimental case and in our modeling, the obstacle, even in absence of the bundle, undergoes an overdamped motion. This conclusion is in agreement with the transient behaviors observed in Reference [18]. Moreover, our model reproduces correctly the ratio between the free oscillation time of the obstacle in the trap and obstacle diffusion times, while the inertial time is somewhat slower than in the experiments, but still two orders of magnitude faster than the diffusion time.

The last important characteristic time is related to chemical reactions at the tips of the filaments. We introduce a chemistry characteristic time $\tau_{\mathrm{chem}}={\tilde{W}}_{0}^{-1}$ for which the standard experimental value ${\tilde{W}}_{0}=1.4s^{-1}$ leads to $\tau_{\mathrm{chem}}/\tau_{D}=2.04\times 10^{4}$. This indicates that the wall diffusion is much faster than the time scale over which an F-actin filament fluctuates in size (Note that the chemical reaction is a very fast event, usually considered instantaneous. Here, $\tau_{\mathrm{chem}}$ gives an estimate of the time interval between two successive reactions). It is useful to mention here that these conditions are precisely assumed to establish the rigid filament velocity-load relation in Equation (1) and its many filament generalization provided by the Mogilner–Oster model ([2,3,4]).

In our simulations, $\tau_{\mathrm{chem}}/\tau_{D}$ is fixed by the parameter *ν*, discussed in Appendix A. We adopt $\nu =7.1\times 10^{4}\tau_{D}^{-1}$, which, using Equation (A21), leads to ${\tilde{W}}_{0}\approx \nu \exp\left(-\beta \epsilon_{0}\right)=0.0844\tau_{D}^{-1}$ to which corresponds the chemical time scale $\tau_{\mathrm{chem}}=11.85\tau_{D}$, hence an acceleration of the chemical rates by a factor $1.7\times 10^{3}$ with respect to experiments. Choosing *ν* corresponding to $\tau_{\mathrm{chem}}/\tau_{D}=2.04\times 10^{4}$ would require $\tau_{\mathrm{chem}}/h\approx 4\times 10^{8}$ time steps between two successive depolymerization reaction events.

As we will show in the next section, the obstacle dynamics in the presence of the F-actin bundle undergoes a motion at equilibrium with a characteristic time, which we denote $\tau_{L}$, roughly 1000 times larger than $\tau_{\mathrm{chem}}$, the largest time discussed so far. This is the signature of the Brownian ratchet mechanism governing the interaction between the chemically-fluctuating bundle and the loaded obstacle. In order to get quantitative information on the obstacle motion, we need to follow the dynamics of the system for many $\tau_{L}$. With the adopted time step *h*, $\tau_{L}$ corresponds to roughly half a billion time steps; therefore, any trajectory should contain several billions of time steps in order to compute $\tau_{L}$. This is the reason why we had to compress considerably the experimentally wide range of characteristic times as discussed in this section.

**Figure 2:**
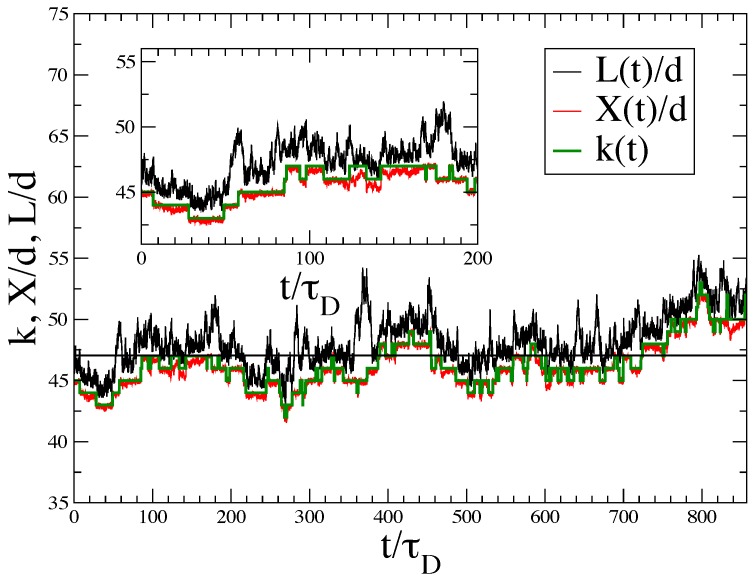
Illustrative molecular dynamics trajectory for a single actin filament in a harmonic trap with trap strength $\kappa_{T}=0.019375kT/d^{2}$ at ${\hat{\rho }}_{1}=2.5$ and $k_{B}T=1$. The green step function gives the number of bonds k(t) of the filament (number of monomers minus one); the red curve shows the normal component of the filament end-to-end vector X(t); and the black curve shows the fluctuating wall position L(t) during a time window corresponding to the wall slowest relaxation time $\tau_{L}\approx 850\tau_{D}$. ${\tilde{W}}_{0}=0.080\tau_{D}^{-1}$ and ${\tilde{U}}_{0}={\hat{\rho }}_{1}{\tilde{W}}_{0}=0.20\tau_{D}^{-1}$ are the bulk depolymerizing and polymerizing rates, respectively. As a result of the wall presence, the global effective polymerization and depolymerization rates obtained by cumulating all successful events where the filament size jumps instantaneously by +1 or −1 are equal to $\approx {\tilde{W}}_{0}$ along an equilibrium trajectory. Over the shown time interval of $t=850\tau_{D}$ one observes indeed ${\tilde{W}}_{0}t=0.08\times 850∼70$ polymerizations and ∼70 depolymerizations.

Figure 2 illustrates the occurrence of several, widely-separated time scales in the system dynamics for the single filament case discussed in the Result section.

## 4. Illustrative Results

We report results from two simulations, one for a single filament ($N_{f}=1$) and one for a homogeneous bundle of $N_{f}=8$ filaments, the latter case being the typical system observed in the in vitro experiments of Reference [18]. Except for the values of $N_{f}$ and $\kappa_{T}$, all experiments are done with the same external parameters, i.e., a transverse area $A=36d^{2}$, a temperature $k_{B}T=1$ and the free monomer chemical potential $\beta \mu_{1}^{*}=-3.6654+\ln2.5=-2.7492$, corresponding to ${\hat{\rho }}_{1}=2.5$. The pressure force due to the free monomers in the second chamber of the optical trap ensemble in Equation (16) is fixed by Equation (19). All internal parameters, described in Section 3 are taken to be similar in the two experiments.

The choice of $\kappa_{T}$ requires some care, and we exploit for guidance the theoretical predictions for rigid filaments, Equations (2) and (4). The non-escaping regime for actin filaments at ${\hat{\rho }}_{1}=2.5$ is limited to an extreme value $L_{\max}=70.2d$ [6]. By choosing $\kappa_{T}=0.019375kT/d^{2}$ for $N_{f}=1$, we expect to be in the condition $\langle L\rangle =47.3d<L_{\max}-3\frac{k_{B}T}{\kappa_{T}}=70.2d-21.6d=48.6d$, which guarantees that the whole range of *L* values probed during the wall fluctuations lies within the non-escaping regime [6]. For $N_{f}=8$, we take $\kappa_{T}=0.275k_{B}T/d^{2}$, which provides 〈L〉≈26.6d with $\sigma_{L}=1.90d$ for rigid filaments (see the Supplemental Material of Reference [6]), again in the good range of *L* values.

As discussed quantitatively in Reference [6], short flexible filaments behave like rigid filaments, while deviations are observed for longer filaments even before entering in the escaping regime. Further dynamical effects of filament flexibility will be discuss in a future publication [7]. We limit the present investigation to nearly rigid cases since our main aim here is to validate the simulation approach against known theories and/or existing experiments.

To generate the initial configurations for our optical-trap simulations, we have first fixed the obstacle wall at the expected average value given by Equation (2) and let the filaments, with initially only two monomers, grow during the dynamics. After a long enough simulation, the system reaches the statistical equilibrium at given ($\mu_{1}^{*},T,L,A$). This state is the initial configuration for a new run with the mobile wall according to the equations of motion (16). The wall position is monitored in time to let the wall equilibrate before starting the production run. This procedure is repeated in parallel for a number of equivalent initial configurations (with only two monomers per filament) with a different random number seed, which ensures that we obtain statistically independent, but equivalent realizations of the dynamics, for the purpose of statistics.

For $N_{f}=1$, we ran 32 independent trajectories for a time interval of $5.6\times 10^{4}\tau_{D}$ per trajectory. The observed wall relaxation time, obtained from the time correlation function of the wall fluctuation, is $\tau_{L}≃950\tau_{D}$; hence, each trajectory covers more than 50 wall relaxation times.

For $N_{f}=8$, we ran 50 independent trajectories of $1.3\times 10^{4}\tau_{D}$. In this case, the observed wall relaxation time is $\tau_{L}≃1400\tau_{D}$; hence, each trajectory covers roughly 10 wall relaxation times.

Figure 2 shows a portion of an equilibrium trajectory for the $N_{f}=1$ case, in a time window of $\tau_{L}\approx 900\tau_{D}$, roughly one wall relaxation time. Chemical reactions are easily detected by the discontinuous variations of the number of bonds k(t) in the filament. The normal component of the filament end-to-end vector X(t) and the instantaneous wall position L(t) show that the wall executes a Brownian ratchet type dynamics, polymerization steps being clearly allowed by natural excursions of the wall towards larger *L* values. Note that in general, X(t)/d is slightly smaller than *k* due to the filament bending. Occasionally, the opposite is seen when the filament is relatively straight and subject to a positive fluctuation of its contour length due to bond stretching.

### 4.1. Static Properties

In Table 3, we report relevant equilibrium averages of the static properties for the bundles. We compute the average trap length 〈L〉 and its distribution, the average size of the bundle $\langle I\rangle =\langle \sum_{n=1}^{N_{f}}j_{n}\rangle /N_{f}$ (a collective property), the average size of the single filament in the bundle $\langle i\rangle =\langle j_{n}\rangle$ (an individual filament property), the average longitudinal component of the end-to-end vector of the single filaments $\langle X\rangle =\langle x_{j_{n}}-x_{1}\rangle$ and the average modulus of the transverse component of the end-to-end vector of the single filaments $\langle R_{⊥}\rangle =\langle \sqrt{{\left(y_{j_{n}}-y_{1}\right)}^{2}+{\left(z_{j_{n}}-z_{1}\right)}^{2}}\rangle$.

**Figure 3:**
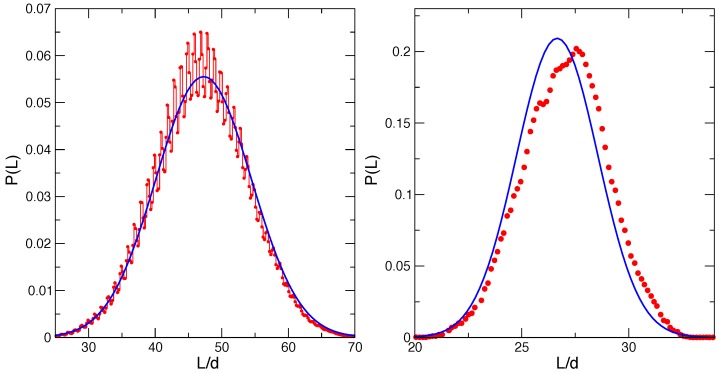
Equilibrium wall distribution P(L) for a single filament ($N_{f}=1$, left panel) and a bundle of eight filaments ($N_{f}=8$, right panel) growing at ${\hat{\rho }}_{1}=2.5$ against a mobile wall subject to a trap-restoring force. The Gaussian curve indicates the equilibrium distribution based on the mean field model of Hill and Kirschner [6].

Figure 3 reports the distributions of the wall position P(L) for the two bundle sizes $N_{f}=1$ (left panel) and $N_{f}=8$ (right panel). In each case, we compare with the theoretical estimate $P\left(L\right)\propto \exp[-\beta \left(\Omega \left(L\right)+\kappa_{T}L^{2}/2\right)]$ based on the bundle free energy $\Omega \left(L\right)=-F_{H}L$ with $F_{H}=N_{f}\left(k_{B}T/d\right)\ln{\hat{\rho }}_{1}$ [6]. We recently demonstrated the statistical mechanics foundations of this Gaussian distribution, with maximum at $L_{H}=F_{H}/\kappa_{T}$ and width $\sigma_{H}=\sqrt{k_{B}T/\kappa_{T}}$. In particular, $F_{H}$ can be shown to be the average force exerted by the bundle of rigid living filaments on a hard mobile wall, within the optical trap grand canonical ensemble and in the continuous limit.

The present filament model has bending flexibility adjusted to F-actin, small contour length compressibility (with amplitude of ≈0.1d) and a discretized contour length with step ≈d. Moreover, the filament-wall interaction is a soft repulsive potential Equation (8) with a wall effective core at $s^{*}=0.846d$ defined by $U^{w}\left(s^{*}\right)=k_{B}T$. In Reference [6], we considered a model of a hard obstacle and discussed the behaviors of a fully-rigid and a flexible (only bending flexibility adjusted to F-actin) model, with a discrete contour length step of *d*. For a single rigid filament, spectacular oscillations on the spacial scales *d* in P(L) were observed as a result of discreteness effects (see Figure 3 of Reference [6]). For the flexible model, they were rounded off to some extent by the flexibility. The features of the distribution in Figure 3 for $N_{f}=1$, for the present model, show a similar behavior, with an expected overall Gaussian shape and oscillations on the scale of ≃d, rounded off by the filament flexibility, filament compressibility and softness of the wall repulsion. In Reference [6] the flexibility was shown to produce an increase of the average force by a few percent, with respect to the rigid case. Our present model provides an average wall position (average force) in agreement with the rigid model results within the present statistical uncertainty. This shows the adequacy of the present model, at least for static equilibrium properties.

The $N_{f}=8$ case in the right panel of Figure 3 shows a much smoother behavior, as expected for a homogeneous bundle. Results for the average trap length and its variance are reported in Table 3. We note that the present bundle model provides a slightly larger average trap length ($\langle L\rangle >L_{H}$) and fluctuation. A similar behavior was observed in Reference [6] for the model with flexible, but incompressible filaments in the presence of a hard wall (see Figure 5a in that reference for a similar case and its Supplemental Material for numerical values of the system with the same external constraints). This observation reinforces our previous finding that flexibility enhances the average bundle force. Furthermore, taking as a reference Hill’s value for the average trap length $L_{H}$, the deviation in the present flexible and compressible filament model in the presence of a soft wall ($\langle L\rangle /L_{H}-1=0.015$) is roughly two-times the deviation of the flexible, but incompressible model with the hard wall ($\langle L\rangle /L_{H}-1=0.006$; see the Supplemental Material of Reference [6]), suggesting that the compressibility and the soft character of the obstacle further enhances the effects of flexibility.

Direct calculations of the average force exerted by the filaments on the wall give $\langle F_{\mathrm{bun}}\rangle =7.4413k_{B}T/d$, $\langle F_{\mathrm{bun}}^{2}\rangle =153.95{\left(k_{B}T/d\right)}^{2}$, in very good agreement with the estimate from $\kappa_{T}\langle L\rangle =7.437\left(k_{B}T/d\right)$, the equivalence being easily proven by statistical mechanics [6].

In Reference [6] for a discrete worm-like chain (WLC) with seeds at $h_{n}$, hitting a hard wall located at *L*, we introduced a crossover index $z_{n}$ by the expression:

(26) $$\begin{array}{c} z_{n}=1+INT\left(\frac{L-h_{n}}{d}\right) \end{array}$$

The filament *n* with size $z_{n}$ (and contour length $L_{c}=\left(z_{n}-1\right)d$) is the longest filament starting at $h_{n}$, which does not hit the wall at *L*. The filament *n* of size $i_{n}$ can then also be specified by a relative index:

(27) $$\begin{array}{c} m=i_{n}-z_{n} \end{array}$$

where m>0 indicates a filament hitting a wall and m≤0 a filament that does not interact directly with the wall.

In the present study, we deal with a slightly different model, as we have a soft repulsive wall that starts at a distance $s_{c}\approx 1.20d$ from the geometrical position of the wall at *L*, and in addition, the contour length is no longer strictly constant. We adopt here the same definitions of $z_{n}$ and *m* in terms of $h_{n}$ and the geometrical wall position *L*, keeping in mind that a filament will now hit the wall strongly when $i_{n}\geq z_{n}$ (m≥0). For the case $i_{n}\leq z_{n}-2$ (m≤−2), the filament (with a fixed contour length of at most $(z_{n}-2)d$) does not interact with the wall, as its tip remains further than 0.8d from the plane at $L-h_{n}-y_{c}$ where the wall repulsion would start to be felt. The small filament contour fluctuations (of the order of 0.1d) would not substantially change the situation. Finally, the case m=−1 is clearly a case of weak interferences with the wall.

**Figure 4:**
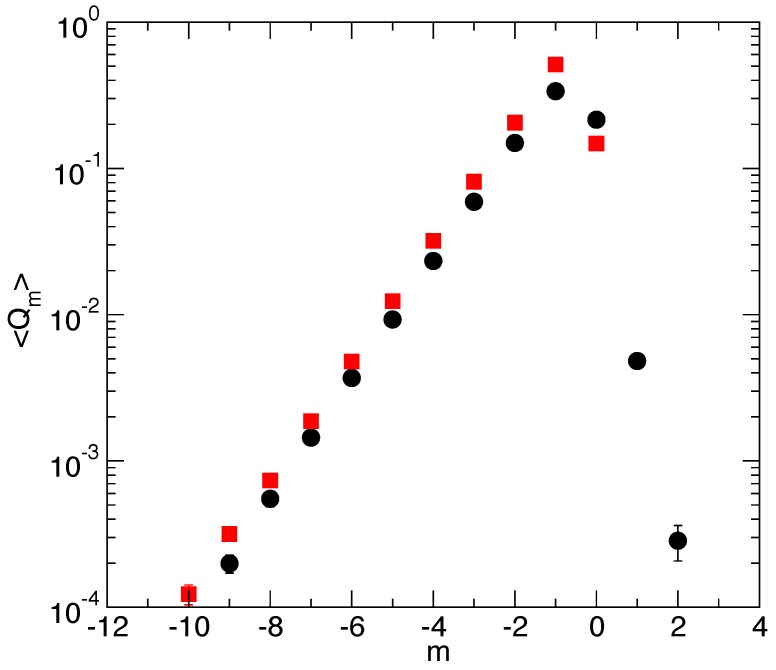
Relative size distribution for filament(s) at ${\hat{\rho }}_{1}=2.5$ pressing against a mobile wall in an optical trap. In black filled circles, the $N_{f}=1$ data with $\kappa_{T}=0.019375kT/d^{2}$ and in red filled squares, the $N_{f}=8$ data with $\kappa_{T}=0.275k_{B}T/d^{2}$. The exponential increase of the distribution yields $\langle Q_{m}\rangle \propto {\hat{\rho }}_{1}^{m}$ with the fit value giving ${\hat{\rho }}_{1}=2.527$ for the $N_{f}=1$ case and ${\hat{\rho }}_{1}=2.525$ for the $N_{f}=8$ case. $\langle Q_{m}\rangle$ values for m>0 for the $N_{f}=8$ case are much smaller than the shown range and, in fact, cannot be determined with precision.

Figure 4 reports the relative size distribution $\langle Q_{m}\rangle$ for the two experiments. We observe a exponential rise $\langle Q_{m}\rangle \propto {\hat{\rho }}_{1}^{m}$ (to a very good accuracy) for negative *m* values up to m=−1 inclusive. The decay of $\langle Q_{m}\rangle$ for m≥0 reflects a strong wall influence and, hence, the decrease towards zero of the wall factor $\alpha (i_{n},L-h_{n})<1$ introduced in References [5,6], and giving $\langle Q_{m}\rangle \propto \alpha \left(z_{n}+m,L-h_{n}\right){\hat{\rho }}_{1}^{m}$. The vertical shift between the two exponential rises reflects the strong difference in the tails for positive *m*, which affects the normalization (for $N_{f}=8$, $\langle Q_{1}\rangle$ is out of scale and not significantly different from zero).

### 4.2. Dynamical Properties

#### 4.2.1. Analysis of Chemical Kinetics

During the experiments, we monitor different counters in which we cumulate information for the $N_{f}$ filaments at each time step *h*. The *m* index of each individual filament is computed, and a counter aiming at the $\langle Q_{m}\rangle$ calculation is increased by one. If a (de)polymerization step is attempted at that time step for a filament of relative size *m*, we add one to a counter of (de)polymerization attempts for a filament index *m*. If a (de)polymerization step is accepted and realized at that time step for a filament of relative size *m*, we add one to a counter of (de)polymerization successful steps for a filament of index *m*. At the end of the run, the chemical events’ counters with index *m* are divided by the $\langle Q_{m}\rangle$ counter and by the time step *h* to get the best estimates of the attempt (de)polymerization rates $U_{m}^{\mathrm{att}}$ and $W_{m}^{\mathrm{att}}$ and of the effective (de)polymerizing rates $U_{m}$ and $W_{m}$. At equilibrium, the total rate of polymerization must be equal to the total rate of depolymerization,

(28) $$\begin{array}{c} \sum_{m=-\infty }^{+\infty }\langle Q_{m}\rangle U_{m}=\sum_{m=-\infty }^{+\infty }\langle Q_{m}\rangle W_{m} \end{array}$$

**Figure 5:**
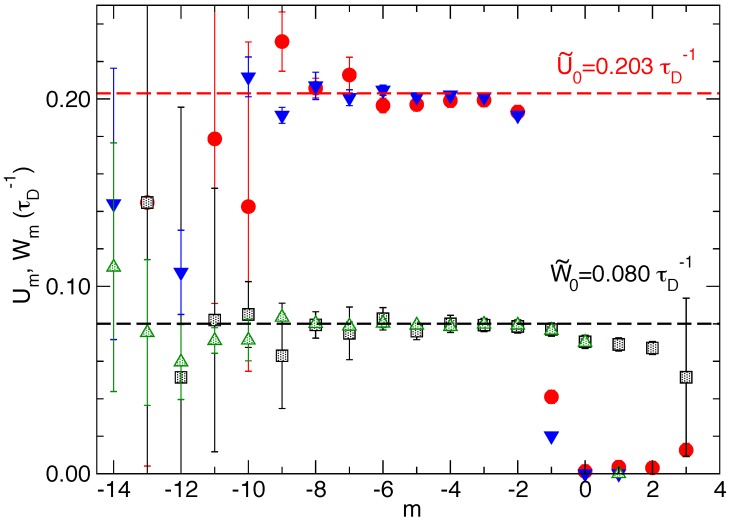
Relative size effective polymerization rate $U_{m}$ (red filled circle for $N_{f}=1$; blue filled triangle for $N_{f}=8$) and effective depolymerization rate $W_{m}$ (black open square for $N_{f}=1$; green open triangle for $N_{f}=8$) for both experiments at the same supercritical state ${\hat{\rho }}_{1}=2.5$ for filaments against a mobile wall subject to a trap-restoring force. The values of the effective bulk (de)polymerization rates correspond to the same plateau value, indicated by a dashed line. We get ${\tilde{W}}_{0}=0.080\tau_{D}^{-1}$ for m<−1 and ${\tilde{U}}_{0}=0.203\tau_{D}^{-1}$ for m<−2. The ratio ${\tilde{U}}_{0}/{\tilde{W}}_{0}=2.54$ is very close to the expected value ${\hat{\rho }}_{1}=2.5$. Statistical noise below m=−10 increases considerably because of the very low number of states sampled during the dynamical simulation.

Figure 5 shows the effective (de)polymerization rates $W_{m},U_{m}$ computed in both experiments ($N_{f}=1,8$). Agreement is observed between the two experiments. Furthermore, the ratio of the two plateau values, corresponding to the bulk (de)polymerization rates, provides the value of 2.54, in tight agreement with the supercritical condition:

(29) $${\hat{\rho }}_{1}=\frac{{\tilde{U}}_{0}}{{\tilde{W}}_{0}}=2.5$$

imposed by fixing the free monomer chemical potential $\mu_{1}^{*}$ with the external reservoir. Attempt rates are given for the $N_{f}=1$ case in the Appendix (see the Figure in Appendix A.2.) with some detailed analysis of their values as a function of *m*.

#### 4.2.2. Wall Relaxation and Related Equilibrium Time Correlation Functions

**Figure 6:**
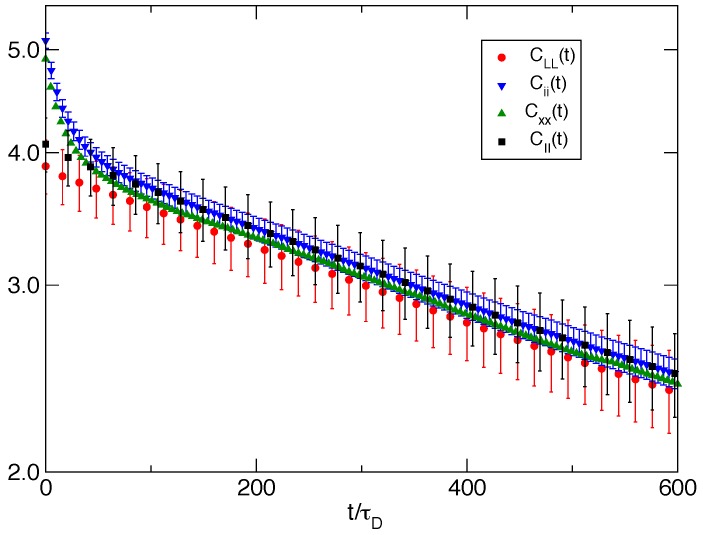
Various related time correlation functions 〈δA(t)δA(0)〉 relative to the $N_{f}=8$ experiment. The main relaxation (red circles) is provided by the wall position A=L, which is fitted by $3.785\exp\left(-t/\tau_{L}\right)$ with $\tau_{L}=1375\tau_{D}$. The relaxation of the longitudinal part of the single filament end-to-end vector A=X (green triangles) is very similar to the wall relaxation, except for an additional relaxation at a short time. The single filament size fluctuations A=i (blues triangles) and the bundle average size fluctuations A=I (black squares) show similar time relaxation curves to, respectively, *X* and the wall position.

In Figure 6 for the bundle of $N_{f}=8$ filaments, we plot the time correlation function of the equilibrium fluctuations of several related properties. The autocorrelation function of wall fluctuations $C_{LL}\left(t\right)=\langle \delta L\left(t\right)\delta L\left(0\right)\rangle$ exhibits a single exponential behavior well represented by $C_{LL}\left(t\right)=3.785\exp\left(-t/\tau_{L}\right)$ with $\tau_{L}=1375\tau_{D}$. A similar single exponential behavior (with a slightly larger zero time value; see Table 3) is followed by the autocorrelation function of the fluctuations of the bundle size $C_{II}\left(t\right)$ proving that the slow relaxation of the wall fluctuation is tightly related to the chemical events and to the Brownian ratchet mechanism induced collectively by the bundle. On the other hand, fluctuations of single filament properties, like the longitudinal component of the single filament end-to-end vector $C_{XX}\left(t\right)$ and the single filament size $C_{ii}\left(t\right)$, exhibit a relaxation with two characteristic times that can be well fitted by a linear combination of exponentials. The slow relaxation time is $\approx \tau_{L}$, while the fast relaxation time is $\tau_{\mathrm{fast}}≃30\tau_{D}$.

For the single filament case, we observe a single exponential relaxation (not shown) with a characteristic time of $\tau_{L}≃950\tau_{D}$.

**Figure 7:**
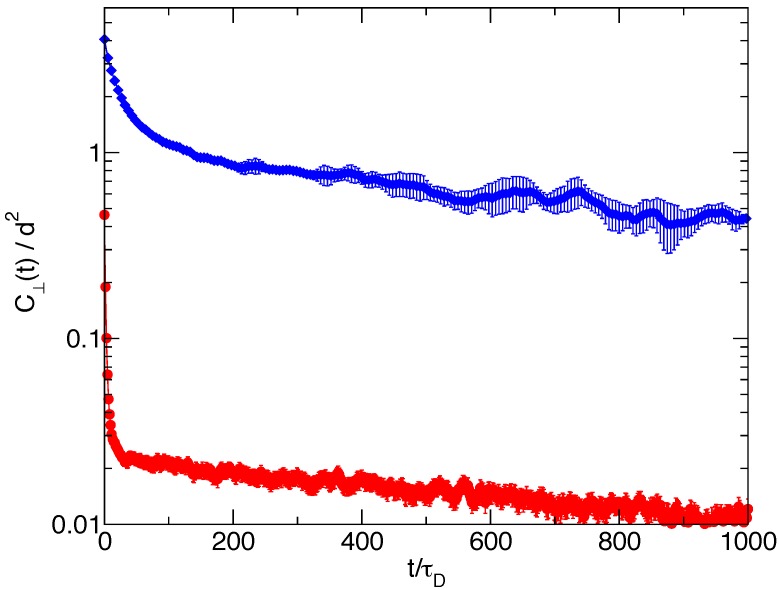
Time correlation function of the fluctuation $\delta R_{⊥}\left(t\right)=R_{⊥}\left(t\right)-\langle R_{⊥}\rangle$ of the modulus $R_{⊥}=\sqrt{{\left(y_{j_{n}}-y_{1}\right)}^{2}+{\left(z_{j_{n}}-z_{1}\right)}^{2}}$ of the transverse part of the end-to-end vector of the single filament of the bundle with $N_{f}=8$ (red circles) and $N_{f}=1$ (blue diamonds).

Finally, in Figure 7, we report for both $N_{f}=1$ and $N_{f}=8$ the autocorrelation function of the fluctuations of the length of the transverse single filament end-to-end vector. Again, a linear combinations of two exponential decays represent well the relaxations, the long relaxation time being again the trap length relaxation time for the respective cases. Quite different values are observed for the two short relaxation times; for $N_{f}=1$, we have $\tau_{\mathrm{fast}}≃25\tau_{D}$, while for $N_{f}=8$, it is $\tau_{\mathrm{fast}}≃2.7\tau_{D}$. This short characteristic time is related to the relaxation of the intramolecular degrees of freedom, and the observed difference between the two cases can be qualitatively justified observing that the eight-filament bundle system has an average trap length quite shorter than the one-filament system, with shorter and more rigid filaments, hence with faster relaxation times. Note that a wide separation between filament equilibration times and the wall characteristic time is at the basis of the adiabatic approximation used in [7] for flexible filaments.

## 5. Conclusions

In this work, we have presented a particle-based model to simulate the dynamics of a grafted bundle of parallel living actin filaments pushing on a mobile wall subject to load. The underlying statistical mechanics framework is the reactive grand canonical ensemble dealing with a two-chambered system with a mobile and loaded partition wall separating a supercritical mixture of G-actin monomers and living flexible F-actin filaments from a pure bulk solution of G-actin. Our goal is to provide a working model to study, e.g., the growth of F-actin filopodia against a resisting membrane.

Here, we specialize the model to represent an optical trap setup, a growing bundle of semi-flexible actin filaments pressing on a wall subject to a restoring force increasing linearly with the wall position *L*. This apparatus allows in particular to measure the bundle polymerizing force [18] (see Figure 1). Our work provides a theoretical tool able to predict quantitatively the link between the wall motion and the bundle’s filament kinetics, the connection empirically described by the velocity-load relationship. In addition, we can also analyze the critical conditions under which the occurrence of filaments escaping laterally, hence no longer participating in the conversion of chemical free energy into work on the loaded wall, can be safely avoided. Our simulation approach is then complementary to optical trap experiments on the actin bundle polymerization forces, experiments that have proven so far to be difficult to perform and to interpret, mainly as a result of the flexible character of the pushing filaments.

Our model actin proteins are diffusing (mesoscopic) point-particles able to assemble/disassemble into linear semi-flexible structures by explicit single monomer (de)polymerization steps, under conditions where the free monomer chemical potential is imposed uniformly in the system. The chemical steps at the filament tips are governed by stochastic rules which fix the bulk (de)polymerizing rates and automatically deal with a realistic influence of the obstacle wall on the chemical rates when the tip is approaching the wall. Although the method works if excluded volume (EV) interactions are considered, the present work is limited to ideal system conditions (no EV) so that monomers interact only with the walls and, when integrated within a linear filament, are subjected to bonding and bending interactions with first and second neighboring monomers.

We have shown that the present mesoscopic approach with explicit consideration of filament bending flexibility allows us to tackle all relevant length scales realistically, in particular the filament length and the filament persistence length. Conversely, given the wide spread of the realistic time scales from the very fast filament bending modes to the slow wall relaxation kinetics induced by the Brownian ratchet mechanism of the pushing bundle, one has to condense the times scales on a narrower range. We have accelerated artificially the chemical reactions by a factor of ≈1700 to make our calculations feasible within a single simulation approach. Despite such an acceleration of one particular process, we have preserved the hierarchy of characteristic times associated with the various dynamical processes taking place within the system.

We illustrated the approach considering a single filament system and a system with a homogeneous bundle of eight filaments. We studied static properties and fluctuation dynamics at equilibrium at the super critical condition ${\hat{\rho }}_{1}=2.5$. The trap strength $\kappa_{T}$ was chosen in each experiment, such that important flexibility effects are avoided, the strategy being to first test the method on cases where a rigid filament approach provides a semi-quantitative, but robust reference.

We have shown that our results are coherent with those predicted theoretically both for a strictly rigid model and for a model of flexible worm-like chains hitting a hard wall in the grand canonical ensemble [6].

The present approach needs further tests in conditions where flexibility effects become more relevant (lower $\kappa_{T}$, longer filaments) and in conditions where the acceleration of the chemistry is reduced to test the influence of the ratio of characteristic times governing chemical steps and wall diffusion in the absence of the bundle. More ambitious developments could envisage the replacement of the rigid obstacle wall by a flexible membrane, the consideration of additional proteins developing branched networks or capping active actin filaments to stop polymerization. Consideration of free monomer concentration inhomogeneities and the distinction between ATP and ADP actin complexes could also be envisaged, showing the rich potential of our model.

## Figures and Tables

**Table 1:** List of parameters in the experimental and model systems.

Symbol	Nature	Experiments	Model	Comment
*d*	monomer size in the filament	2.7 nm	1	unit of length
$k_{B}T$	thermal energy	$4.142\times 10^{-21}$ J	1	unit of energy
$\tau_{D}=\zeta^{\prime }d^{2}/k_{B}T$	wall diffusion characteristic time	$3.5\times 10^{-5}$ s	1	unit of time
$k_{B}T/d^{2}$	scale of the force constant	0.57 pN/nm	1	conversion factor
$M/(\tau_{D}^{2}k_{B}T/d^{2})$	free monomer mass	$10^{-10}$	$3.556\times 10^{-3}$	$M^{\exp}=6.98\times 10^{-23}$ kg
$M_{w}/\left(\tau_{D}^{2}k_{B}T/d^{2}\right)$	obstacle mass	$0.59\times 10^{-2}$	$1.0667\times 10^{-2}$	$M_{w}^{\exp}=4.2\times 10^{-15}$ kg
$\zeta^{\prime }/\left(\tau_{D}k_{B}T/d^{2}\right)$	solvent friction on the obstacle	1	1	$\zeta_{\exp}^{\prime }=1.9\times 10^{-8}$ Ns/m
$\zeta (\tau_{D}k_{B}T/d^{2})$	solvent friction on free monomers	$2.76\times 10^{-3}$	0.5	$\zeta_{\exp}=5.5\times 10^{-11}$ Ns/m
$L_{p}/d$	persistence length of F-actin	5370	5370	$L_{p}=14.5\mu m$
〈L〉/d	typical filament length	20–100	20–100	–
${\hat{\rho }}_{1}$	reduced free monomer density	1.5–4.0	2.5	connected to chemical potential $\mu_{1}^{*}$
$K_{0}d^{-3}$	bulk chemical equilibrium constant	–	39.07144	critical density $\rho_{1c}=1/K_{0}$

**Table 2:** List of characteristic time scales of the experimental and model systems. GC, grand canonical ensemble.

Symbol	Nature	Experiments	Model	Comment
$\tau_{s}/\tau_{D}$	stretching mode	$3.69\times 10^{-7}$	$5.9\times 10^{-3}$	stiffest stretching mode
$\tau_{b}/\tau_{D}$	bending mode	$6.06\times 10^{-7}$	$3.6\times 10^{-3}$	stiffest bending mode
$\tau_{\mathrm{fm}}^{\mathrm{in}}/\tau_{D}$	monomer inertial time	$3.6\times 10^{-8}$	$7.1\times 10^{-3}$	–
$\tau_{\mathrm{fm}}/\tau_{D}$	monomer diffusion time	$3.14\times 10^{-3}$	0.58	actin radius = 2.9 nm/same water solvent
$\nu_{\mathrm{GC}}\tau_{D}$	monomer insertion/deletion attempt rate	–	187.5	exchange rate with GC reservoir
$\tau_{w}^{\mathrm{in}}/\tau_{D}$	wall inertial time	$6.37\times 10^{-3}$	$1.1\times 10^{-2}$	for trap in absence of actin
$\tau_{T}/\tau_{D}$	trap natural period	4.1	4.7–1.2	for typical exp.value
$h/\tau_{D}$	time step	–	$5.33\;10^{-5}$	–
$\nu \tau_{D}$	attempted reaction frequency	–	$7.1\times 10^{4}$	tuning absolute reaction rates
$\tau_{\mathrm{chem}}/\tau_{D}$	chemistry characteristic time	$2.041\times 10^{4}$	12.5	$\tau_{\mathrm{chem}}={\tilde{W}}_{0}^{-1}={\tilde{U}}_{0}^{-1}{\hat{\rho }}_{1}$

**Table 3:** Equilibrium averages of static properties of the systems. $\langle N_{1}\rangle$ indicates the number of free monomers in the system, 〈L〉 the average position of the wall, $L_{H}$ the wall position according to Equation (2), 〈I〉 the average size of the bundle and 〈i〉 the average size of a single filament and 〈X〉 and $\langle R_{⊥}\rangle$ the longitudinal and transverse components of the end-to-end vector of the single filaments, respectively.

Property	$N_{f}=1$	$N_{f}=8$
$\kappa_{T}d^{2}/k_{B}T$	0.019375	0.275
$\langle N_{1}\rangle$	105.6(6)	58.4(3)
$\sigma_{N_{1}}$	18.7	8.9
〈L〉/d	47.1(2)	27.05(8)
$\sigma_{L}/d$	6.97	1.97
$L_{H}\left(d\right)$	47.29	26.65
$\sigma_{H}/d$	7.184	1.9069
〈I〉	46.3(2)	26.14(8)
$\sigma_{I}$	7.13	2.02
〈i〉	46.3(2)	26.14(8)
$\sigma_{i}$	7.13	2.28
〈X〉/d	45.1(2)	25.6(4)
$\sigma_{X}$	7.01	2.21
$\langle R_{⊥}\rangle /d$	3.2(2)	1.28(4)
$\sigma_{⊥}$	2.02	0.68

**Figure A1:**
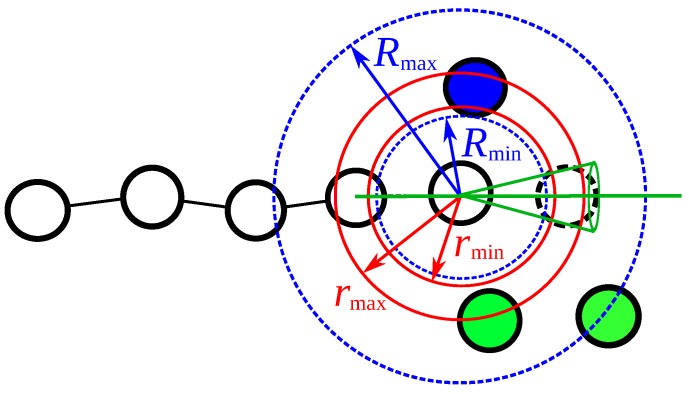
State *J*: The depolymerized state for filament *n* with size $j_{n}$ where the reacting monomer of index *k* shown in blue is free, but is necessarily located within the spherical layer $\Omega_{s}\left(n,j_{n}\right)$ limited by spheres of radii $R_{\min}$ and $R_{\max}$ centered on monomer $\left(n,j_{n}\right)$ shown with dashed-blue lines (see Equations (A1) and the related text for details). The dashed sphere represents the position of the reacting monomer after a possible polymerization ending with configuration in state *I* (see Figure A2). Green filled circles represent other free monomers in the environment, while connected full circles represent the grafted filament end monomers in the depolymerized case. Reproduced from J. Chem. Phys. 136, 114901 (2012) [17]; http://dx.doi.org/10.1063/1.3694672, with the permission of AIP Publishing.

**Figure A2:**
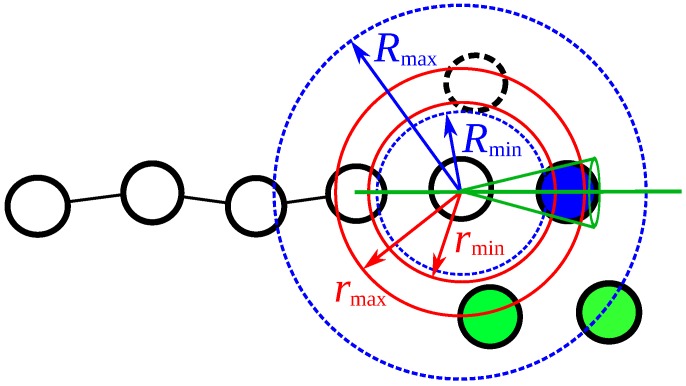
State *I*: The polymerized state for filament *n* with size $\left(j_{n}+1\right)$ where the reacting monomer with intrinsic index *k* shown in dark blue is the tip of the filament and is, necessarily, located in a low intramolecular energy portion of space $\Omega_{c}\left(n,j_{n}+1\right)$ resulting from the intersection of a conic volume (shown in green) of angle $\theta_{\max}$ and a spherical layer (shown in red) limited by spheres of radii $r_{\min}$ and $r_{\max}$ (see Equation (A2) and the related text for details). The dashed sphere represents the position of the reacting monomer after of a possible depolymerization ending with configuration in state *J* (see Figure A1). Reproduced from J. Chem. Phys. 136, 114901 (2012) [17]; http://dx.doi.org/10.1063/1.3694672, with the permission of AIP Publishing.

**Figure A3:**
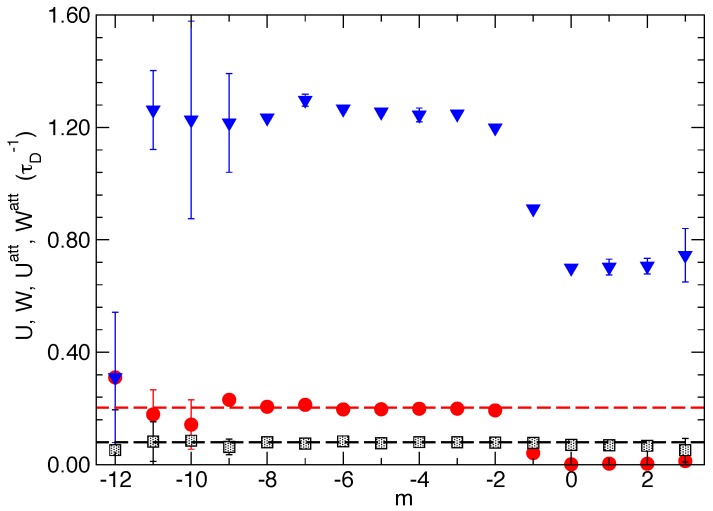
Relative size polymerization and depolymerization rates for the $N_{f}=1$ case at ${\hat{\rho }}_{1}=2.5$. The comparison between the attempt rates $U_{m}^{\mathrm{att}}$ (blue triangles) and $W_{m}^{\mathrm{att}}$ (gray squares) and the effective rates $U_{m}$ (red circles) and $W_{m}$ (superimposed to $W_{m}^{\mathrm{att}}$).

## Appendix A. Single Monomer (De)Polymerization Steps within the Filament-Free Monomer Mixture

### Appendix A.1. Description of the (De)Polymerizing Steps

During a depolymerizing step, the tip monomer of a grafted filament can detach and diffuse away as a free monomer. Reversely, during the polymerizing step, a free monomer having diffused close to a filament’s tip can be captured and becomes the new tip monomer of the filament whose size increases by one unit. This Appendix details when and how these reactions are realized during the dynamical simulation, gives a formal justification of the procedure and provides some useful explicit estimates of single filament (de)polymerizing rates for ideal conditions.

For a polymerization or a depolymerization to take place at the tip of filament *n*, we request the necessary (but not sufficient) condition that the arriving free monomer or the detaching tip monomer satisfies a geometrical criteria, namely that the monomer lies in a well-defined portion of space, $\Omega_{s}\left(n,j_{n}\right)$ for polymerization and $\Omega_{c}\left(n,j_{n}\right)$ for depolymerization, respectively, defined as follows.

- $\Omega_{s}\left(n,j_{n}\right)$ is a spherical layer centered at the location of the tip monomer $\left(n,j_{n}\right)$ with volume:
  (A1) $$V_{s}=\frac{4\pi }{3}\left(R_{\max}^{3}-R_{\min}^{3}\right)$$
  having interior and exterior radii $R_{\min}=0.8909d$ and $R_{\max}=2d$, respectively (These bounds were kept unchanged with respect to [17], where they were motivated by the presence of EV interactions modeled as shifted Lennard–Jones potentials. In the present case where EV are not included, these parameters are merely an arbitrary choice for defining the tip’s volume of capture for diffusing free monomers). Any free monomer located within $\Omega_{s}\left(n,j_{n}\right)$ at time *t* is defined as a reactive free monomer with respect to filament *n* at time *t*. Note that several distinct free monomers can be reactive with the same filament’s tip and that a single free monomer can be reactive with respect to the tips of several filaments.
- $\Omega_{c}\left(n,j_{n}\right)$ is centered on the point where, for a given microscopic configuration of the $j_{n}-1$ first monomers, both the vibrational part of the bonding potential and the bending vibrational potential energies involving the tip monomer $\left(n,j_{n}\right)$ are zero. This point is located at a distance r=d from monomer $\left(n,j_{n}-1\right)$ and is collinear with the bond axis *u* connecting monomers $\left(n,j_{n}-2\right)$ and $\left(n,j_{n}-1\right)$. The portion of space $\Omega_{c}\left(n,j_{n}\right)$ is delimited in space by a spherical shell centered on monomer $\left(n,j_{n}-1\right)$ with extreme radii $r_{\min}$ and $r_{\max}$ and by a cone with apex at the same monomer $\left(n,j_{n}-1\right)$ location, with symmetry axis *u* and with an angle opening $\theta_{\max}$, where all of these *r* and *θ* extreme values correspond to the distances or angles for which the purely intramolecular vibrational term in Equation (5) or in Equation (6) is equal to $3k_{B}T$. This portion of space has a volume of:
  (A2) $$V_{c}=\frac{2\pi }{3}\left(1-\cos\theta_{\max}\right)\left(r_{\max}^{3}-r_{\min}^{3}\right).$$
  A tip monomer $\left(n,j_{n}\right)$ will be defined as reactive at time *t* if it lies within $\Omega_{c}\left(n,j_{n}\right)$, which will be the case most of the time along equilibrium trajectories.

We request that the reactive steps satisfy the micro-reversibility condition at equilibrium:

(A3) π(J)P(J→I)=π(I)P(I→J)

where *J* and *I* are the two microscopic states separated by the reversible single step (de)polymerization reaction, $\pi \left(J\right)\propto e^{-\beta u(J)}$ is the equilibrium Boltzmann weight of state *J* and P(J→I) represents the conditional probability to go from state *J* to state *I*. As in the usual Metropolis method, the conditional probability is split into the product of two probabilities P(J→I)=T(J→I)A(J→I), an a priori sampling probability T(J→I) and an acceptance probability A(J→I). The two distinct states are more precisely defined by the following two processes:

- The polymerization step: Consider the filament *n* with $j_{n}$ monomers in a given microscopic configuration and a free monomer located at some specific position $r_{s}\in \Omega_{s}\left(n,j_{n}\right)$, and consider the process of sampling new coordinates for this free monomer $r_{c}\in \Omega_{c}\left(n,j_{n}+1\right)$ and adding the necessary intramolecular interactions in order to make this monomer the new tip of an extended filament of size $j_{n}+1$. We denote *J* the initial microscopic state and *I* the final state, which are illustrated respectively in Figure A1 and Figure A2;
- The depolymerization step: Consider the same filament *n* with $j_{n}+1$ monomer in the same microscopic configuration (state *I*), and consider the process of moving the tip monomer at its original position prior to the polymerization step $r_{s}\in \Omega_{s}\left(n,j_{n}\right)$ and removing the intrafilament interactions involving monomer $j_{n}+1$. The microscopic state has changed from *I* into *J* in this case.

Given the reactive state *J* (*I*), the a priori probability of state *I* (*J*) is the product of the probability to attempt the reaction and the probability to generate the new position in the final state. We have defined the attempt rates $\nu_{\mathrm{pol}}$ and $\nu_{\mathrm{dep}}$ as:

(A4) $$\begin{array}{cc} \nu_{\mathrm{pol}} & =\nu \frac{V_{c}}{V_{s}+V_{c}} \end{array}$$

(A5) $$\begin{array}{cc} \nu_{\mathrm{dep}} & =\nu \frac{V_{s}}{V_{s}+V_{c}}\exp\left(-\beta \epsilon_{0}\right) \end{array}$$

where each rate is proportional to the volume of the region where the new coordinates will be located and *ν* is a rescaling free parameter with the dimension of inverse time (related to an adjustable energy barrier of the (de)polymerization reaction). In Equation (A5), $\epsilon_{0}$ is the bonding energy term of Equation (5), which acts as a penalty for the depolymerization rate (it is related to the difference between the two energy minima at the two sides of the barrier).

For each microscopic configuration of the system generated during the dynamics at discrete time $t_{l}=l\;h$, all reactive states are systematically detected. We assume that the times at which the possible reactions occur are independent of each other and distributed according to a Poisson law $P_{\alpha }\left(t\right)=\nu_{\alpha }e^{-\nu_{\alpha }t}$ (α=pol,dep). The probability to attempt a reaction during the next time step *h* is the cumulative function of $P_{\alpha }\left(t\right)$, namely $F_{\alpha }\left(h\right)=\left(1-e^{-\nu_{\alpha }h}\right)$. To decide whether to attempt a given reaction, we extract a uniformly-distributed random number and compare it with $F_{\alpha }\left(h\right)$ of that specific reaction. Given the values of *h* and of the rates $\nu_{\alpha }$, in practice, $F_{\alpha }\left(h\right)\approx h\nu_{\alpha }\ll 1$, so that in the majority of cases, no reaction is attempted for all reactive monomers, and the dynamical integration algorithm is applied once more to the unaffected microscopic configuration. From time to time, one event is selected, and that unique reaction is attempted by sampling the new location of the reacting monomer uniformly within the target relevant reacting portion of space, defined by the nature of the transition. Therefore, the a priori conditional probabilities are:

(A6) $$\begin{array}{ccc} T_{\mathrm{pol}}\left(J\to I\right) & = & h\nu_{\mathrm{pol}}\frac{1}{V_{c}}=\frac{h\nu }{V_{s}+V_{c}} \end{array}$$

(A7) $$\begin{array}{ccc} T_{\mathrm{dep}}\left(I\to J\right) & = & h\nu_{\mathrm{dep}}\frac{1}{V_{s}}=\frac{h\nu }{V_{s}+V_{c}}e^{-\beta \epsilon_{0}} \end{array}$$

As in the usual Metropolis algorithm, the detailed balance condition Equation (A3) is satisfied by [27]:

(A8) $$\begin{array}{ccc} A_{\mathrm{pol}}\left(J\to I\right) & = & \mathrm{Min}\left[1;\frac{\pi \left(I\right)T_{\mathrm{dep}}\left(I\to J\right)}{\pi \left(J\right)T_{\mathrm{pol}}\left(J\to I\right)}\right]=\mathrm{Min}\left[1;\exp\{-\beta \left[u\left(I\right)+\epsilon_{0}-u\left(J\right)\right]\}\right] \end{array}$$

(A9) $$\begin{array}{ccc} A_{\mathrm{dep}}\left(I\to J\right) & = & \mathrm{Min}\left[1;\frac{\pi \left(J\right)T_{\mathrm{pol}}\left(\to I\right)}{\pi \left(I\right)T_{\mathrm{dep}}\left(I\to J\right)}\right]=\mathrm{Min}\left[1;\exp\{-\beta \left[u\left(J\right)-u\left(I\right)-\epsilon_{0}\right]\}\right] \end{array}$$

which is the usual condition on the energy difference except for the term $\epsilon_{0}$. Note that it appears in both equations with the same sign as u(I). The state *I* differs from *J* because it has one more bond with associated bonding energy Equation (5), where a $-\epsilon_{0}$ term cancels the analogous term in both Equations (A8) and (A9) and associated bending energy Equation (6). Moreover, the values of non-bonding interactions (interaction with the wall and excluded volume interactions when present) involving the displaced monomer are different between the two states. Note that a different, but equally correct algorithm could avoid the penalty term $\exp(-\beta \epsilon_{0})$ in the depolymerization attempt rate and let it in the final acceptance probability. In this case, however, depolymerization events would be sampled much more frequently, but also rejected more frequently with a loss of efficiency of the procedure.

During a chemical step, we do not advance the time of the dynamics, no matter if the reaction is accepted or rejected. Therefore, we treat reactions as instantaneous processes occurring always at the beginning of the generic time step. This is a possible way of coupling normal dynamics with MC steps. A possible justification is that during a chemical event, at most one particle is displaced while the others are still.

### Appendix A.2. Prediction of Macroscopic Rates for the Ideal System

It is instructive to consider the ideal mixture treatment of the (filaments + free monomers) reactive system with the aim to provide computable expressions for the rates $U_{m}$ and $W_{m}$ introduced in Section 4.2.1. For the sake of simplicity in the notation, we limit our derivation to the single filament case; the extension to the multi-filament system is straightforward for non-interacting filaments. Let us consider the filament in state *J* with *j* monomers corresponding to m=j−z (here, $h_{n}=0$) that polymerize to the state *I* with j+1 monomers, hence to m+1 (moving its tip closer to the wall). Because the filaments are rather rigid and the obstacle is a plane, these processes take place most probably in a specific slab parallel to the wall. The rate at which such a process progresses can be written as the product of three different terms: (1) the number of free monomers available for the reaction $N_{r}\left(m\right)$; (2) the rate of the single polymerization event $\nu_{\mathrm{pol}}$ given in Equation (A4); (3) a global acceptance for the proposed processes $A_{p}\left(m\right)$ defined below. The number of reactive monomers $N_{r}\left(m\right)$ in the slab of index *m* is the integral over $\Omega_{s}\left(m\right)$ of the free monomer density profile ${\tilde{\rho }}_{1}\left(r\right)$. Near the wall (m≥−2), the density profile is modified by the repulsive interaction with the wall, and for an ideal system (no particle correlations), it takes the form ${\tilde{\rho }}_{1}\left(r\right)=\rho_{1}\exp\left[-\beta U^{w}\left(r\right)\right]$ where $U^{w}$ is given in Equation (8). Therefore, we can write:

(A10) $$\begin{array}{ccc} U_{m} & = & U_{m}^{\mathrm{att}}\;A_{p}\left(m\right) \end{array}$$

(A11) $$\begin{array}{ccc} U_{m}^{\mathrm{att}} & = & N_{r}\left(m\right)\nu_{\mathrm{pol}}=\frac{V_{c}}{V_{c}+V_{s}}\;\nu \;\int_{\Omega_{s}\left(m\right)}dr\;{\tilde{\rho }}_{1}\left(r\right) \end{array}$$

(A12) $$\begin{array}{ccc} A_{p}\left(m\right) & = & \frac{\int_{\Omega_{s}\left(m\right)}dr_{J}\;{\tilde{\rho }}_{1}\left(r_{J}\right)\;\int_{\Omega_{c}\left(m+1\right)}dr_{I}\;\mathrm{Min}\left[1;e^{-\beta \left[\epsilon_{\mathrm{vib}}\left(r_{I}\right)+U^{w}\left(r_{I}\right)-U^{w}\left(r_{J}\right)\right]}\right]}{V_{c}\;\int_{\Omega_{s}\left(m\right)}dr\;{\tilde{\rho }}_{1}\left(r\right)} \end{array}$$

where $r_{J}$ and $r_{I}$ represent the position of the reactive monomer in states *J* and *I*, respectively, and $\epsilon_{\mathrm{vib}}\left(r_{I}\right)$ represents the vibrational contribution to the energy from the additional bond in state *I*.

A similar expression can be cast for the depolymerization rate $W_{m}$, where configurations in state *I* with *j* monomers, corresponding to m=j−z, go to state *J* with j−1 monomers, corresponding to m−1. In this case, however, the number of reactive monomers must be replaced by the number of reactive tips $N_{t}\left(m\right)$, i.e., the fraction of tip states with large enough statistical weight according to our definition above:

(A13) $$N_{t}\left(m\right)=\frac{\int_{\Omega_{c}\left(m\right)}dr_{I}\;\exp\left[-\beta \left(\epsilon_{\mathrm{vib}}\left(r_{I}\right)+U^{w}\left(r_{I}\right)\right)\right]}{\int_{V}dr_{I}\;\exp\left[-\beta \left(\epsilon_{\mathrm{vib}}\left(r_{I}\right)+U^{w}\left(r_{I}\right)\right)\right]}$$

where *V* is the volume of the entire system, and the wall interaction term ensures that tip positions are confined in the correct region of space. In analogy with the polymerization process above, we can write:

(A14) $$\begin{array}{ccc} W_{m} & = & W_{m}^{\mathrm{att}}\;A_{d}\left(m\right) \end{array}$$

(A15) $$\begin{array}{ccc} W_{m}^{\mathrm{att}} & = & N_{t}\left(m\right)\nu_{\mathrm{dep}}=\nu \frac{V_{s}}{V_{s}+V_{c}}\;e^{-\beta \epsilon_{0}}\;N_{t}\left(m\right) \end{array}$$

(A16) $$\begin{array}{ccc} A_{d}\left(m\right) & = & \frac{1}{V_{s}}\;\frac{\int_{\Omega_{c}\left(m\right)}dr_{I}\;e^{-\beta (\epsilon_{\mathrm{vib}}\left(r_{I}\right)+U^{w}\left(r_{I}\right))}\;\int_{\Omega_{s}\left(m-1\right)}dr_{J}\;\mathrm{Min}\left[1;e^{-\beta \left[U^{w}\left(r_{J}\right)-U^{w}\left(r_{I}\right)-\epsilon_{\mathrm{vib}}\left(r_{I}\right)\right]}\right]}{\int_{\Omega_{c}\left(m\right)}dr_{I}\;e^{-\beta (\epsilon_{\mathrm{vib}}\left(r_{I}\right)+U^{w}\left(r_{I}\right))}} \end{array}$$

Those expressions becomes particularly illuminating when m≤−3, i.e., far from the confining wall, and the wall potential vanishes. In that case, both $U_{m}$ and $W_{m}$ become independent of *m* and equal to their corresponding bulk values ${\tilde{U}}_{0}$ and ${\tilde{W}}_{0}$, respectively. Far from the wall, the free monomer density profiles become constant and equal to $\rho_{1}$ and $N_{r}\left(m\right)=V_{s}\rho_{1}$. The integral over $r_{J}$ in the numerator of Equation (A12) cancels the one in the denominator. Moreover, the Min[⋯] operator in Equation (A12) always selects the exponential since the vibrational energy, the only surviving term, is always positive. As for the depolymerization rate, $N_{t}\left(m\right)$ still presents the ratio of two integrals, while the expression of $A_{d}\left(m\right)$ Equation (A16) becomes trivial since now Min[⋯]=1, again, because of the sign of the vibrational energy. We have:

(A17) $$\begin{array}{cc} {\tilde{U}}_{0} & =\rho_{1}\nu \frac{V_{s}}{V_{s}+V_{c}}\;\;\int_{\Omega_{c}}dr\exp\left(-\beta \epsilon_{\mathrm{vib}}\left(I\right)\right) \end{array}$$

(A18) $$\begin{array}{cc} {\tilde{W}}_{0} & =\nu \;\frac{V_{s}}{V_{s}+V_{c}}\;\;e^{-\beta \epsilon_{0}}\;\;\frac{\int_{\Omega_{c}}dr\;\exp\left(-\beta \epsilon_{\mathrm{vib}}\left(I\right)\right)}{\int_{V}dr\;\exp\left(-\beta \epsilon_{\mathrm{vib}}\left(I\right)\right)} \end{array}$$

(A19) $$\begin{array}{cc} \frac{{\tilde{U}}_{0}}{{\tilde{W}}_{0}} & =\rho_{1}\;e^{\beta \epsilon_{0}}\;\int_{V}dr\;e^{-\beta \epsilon_{\mathrm{vib}}\left(I\right)}=\rho_{1}K_{0}={\hat{\rho }}_{1} \end{array}$$

where the identity $K_{0}=e^{\beta \epsilon_{0}}\int_{V}dr\;\exp\left(-\beta \epsilon_{\mathrm{vib}}\left(I\right)\right)$, following from the definition of $K_{0}$ in Equation (9), was derived in Equation (27) of [17]. We note that $V_{s}\gg V_{c}$, and the two integrals in Equation (A18) are roughly equal, since $\Omega_{c}$ is the portion of space contributing most to the integral in the denominator for the added bond; we can estimate the bulk (de)polymerization rates as:

(A20) $$\begin{array}{cc} {\tilde{U}}_{0} & \approx \nu \rho_{1}K_{0}\exp\left(-\beta \epsilon_{0}\right) \end{array}$$

(A21) $$\begin{array}{cc} {\tilde{W}}_{0} & \approx \nu \exp(-\beta \epsilon_{0}) \end{array}$$

We have computed numerically $U_{m}^{\mathrm{att}},W_{m}^{\mathrm{att}}$ and $U_{m},W_{m}$ using Equations (A11), (A15), (A10) and (A14) during our calculations, averaging also over the specific position of the reactive tip in state *J* at fixed *m* and over the wall fluctuations in our optical trap experiment. Results for the single filament system are shown in Figure A3 for both the attempt rates and the effective rates (already shown in Figure 5). We observe as $U_{m}^{\mathrm{att}}$ is roughly constant and equal to 1.2 far from the wall (m≤−3), a value in agreement with $V_{s}V_{c}/\left(V_{s}+V_{c}\right)\rho_{1}\nu$ as indicated by Equation (A17). Closer to the wall, $U_{m}^{\mathrm{att}}$ decreases roughly linearly between m=−2 and m=0, where it reaches a new constant value around 0.75. This decrease is therefore the combined effect of reducing the available volume of the spherical shell of the tip when approaching the wall and also of reducing the free monomer density because of the repulsion with the wall. As better seen in Figure 5, the final polymerization rate for m≥0 vanishes because no more polymerizations are accepted. Conversely, $W_{m}$ and $W_{m}^{\mathrm{att}}$ are always very close to each other.

## Appendix B. Modeling Constant Free Monomers’ Chemical Potential

We apply the general MC sampling algorithm [24] to the system confined by two walls with fluctuating distance *L* between them and subject to a reservoir of free monomers at chemical potential $\beta \mu_{1}^{*}=-3.6654+\ln{\hat{\rho }}_{1}$ where the first term is the value of the critical chemical potential for our model (see Equation (23)).

Similarly to the implementation of the chemical reactions, we assign a fixed rate of particle exchange with the reservoir $\nu_{\mathrm{GC}}$ (this is the free parameter of our calculation). Along the main dynamical trajectory, the probability to have a particle exchange within the next time step is $h\nu_{\mathrm{GC}}$; hence, prior to each integration step, we compare a random number $r_{1}$, uniformly distributed in [0;1], with such a probability. If $r_{1}\leq h\nu_{\mathrm{GC}}$, a particle exchange is attempted. In this case, we decide randomly whether the exchange move is an insertion or a deletion of a free monomer. At any selected absolute time *t* where such moves are attempted, the system has $N_{1}\left(t\right)$ free monomers and an instantaneous volume V(t)=AL(t).

If the addition of one monomer is selected, a random position is sampled homogeneously in *V*. Optionally (this is what we followed in our applications), we refuse a priori the addition if the new particle turns out to be immediately reactive, that is if it falls in any reactive portion of space $\Omega_{s}\left(n,j_{n}\right)$ of a filament tip (see Appendix B). The new particle, if the optionally test is negative, is accepted and then inserted at the attempted location with a probability:

(B1) $$\begin{array}{cc} A_{\mathrm{GC}}\left(N_{1}⟶N_{1}+1\right) & =\mathrm{Min}\left[1,\frac{V/d^{3}}{N_{1}+1}e^{\beta [\mu_{1}^{*}-\epsilon_{\mathrm{add}}^{\mathrm{ev}}-\epsilon_{\mathrm{add}}^{w}]}\right] \end{array}$$

where $\epsilon_{\mathrm{add}}^{\mathrm{ev}}$ and $\epsilon_{\mathrm{add}}^{w}$ are, respectively, the EV interaction (with all other monomers in the general case) and the wall energy of the extra free monomer at the attempted location.

If the deletion of one free monomer is selected, one existing free monomer among the $N_{1}\left(t\right)$ ones is selected at random. Optionally, if reactive monomers are not allowed for exchanges with the bath, the removal step is a priori refused if the selected monomer is reactive. Then, if the optionally test is negative, the removal of the selected particle from the set of free monomers is accepted with probability:

(B2) $$\begin{array}{cc} A_{\mathrm{GC}}\left(N_{1}⟶N_{1}-1\right) & =\mathrm{Min}\left[1,\frac{N_{1}}{V/d^{3}}\exp-\beta [\mu_{1}^{*}+\epsilon_{\mathrm{rem}}^{\mathrm{ev}}+\epsilon_{\mathrm{rem}}^{w}]\right] \end{array}$$

where $\epsilon_{\mathrm{rem}}^{\mathrm{ev}}$ and $\epsilon_{\mathrm{rem}}^{w}$ are, respectively, the EV interaction (with all other monomers) and the wall energy of the selected free monomer.

If the attempted change in $N_{1}$ is rejected, the dynamics proceeds as it would have done for a normal time step. In case of the acceptance of the exchange move, the number of free monomers is altered, and the usual algorithm is applied to the new set of $N_{1}+1$ or $N_{1}-1$ free monomers. As for the chemical reactions, these exchange processes are considered to be instantaneous and occurring at the beginning of the time step.
